# Sublethal doxorubicin promotes migration and invasion of breast cancer cells: role of Src Family non-receptor tyrosine kinases

**DOI:** 10.1186/s13058-021-01452-5

**Published:** 2021-07-27

**Authors:** Samia Mohammed, Achraf A. Shamseddine, Benjamin Newcomb, Ronald S. Chavez, Tyler D. Panzner, Allen H. Lee, Daniel Canals, Chioma M. Okeoma, Christopher J. Clarke, Yusuf A. Hannun

**Affiliations:** 1 Department of Biochemistry and Cell Biology, Stony Brook University, Stony Brook, NY 11794- 8430 USA; 2 Stony Brook University Cancer Center, MART Level 9, Stony Brook University, Stony Brook, NY 11794-8430 USA; 3 Department of Medicine, Stony Brook University, Health Science Center, Hospital Pavilion Level 5, Stony Brook, NY 11794-8430 USA; 4 Department of Pharmacological Sciences, Stony Brook University, Stony Brook, NY 11794-8430 USA; 5 The Northport Veterans Affairs Hospital, Northport, NY 11768 USA

**Keywords:** Src Family Kinases, Fyn, Yes, Doxorubicin, p53, ATR, Dasatinib, Breast cancer

## Abstract

**Background:**

Doxorubicin (Dox) is a widely used chemotherapy, but its effectiveness is limited by dose-dependent side effects. Although lower Dox doses reduce this risk, studies have reported higher recurrence of local disease with no improvement in survival rate in patients receiving low doses of Dox. To effectively mitigate this, a better understanding of the adverse effects of suboptimal Dox doses is needed.

**Methods:**

Effects of sublethal dose of Dox on phenotypic changes were assessed with light and confocal microscopy. Migratory and invasive behavior were assessed by wound healing and transwell migration assays. MTT and LDH release assays were used to analyze cell growth and cytotoxicity. Flow cytometry was employed to detect cell surface markers of cancer stem cell population. Expression and activity of matrix metalloproteinases were probed with qRT-PCR and zymogen assay. To identify pathways affected by sublethal dose of Dox, exploratory RNAseq was performed and results were verified by qRT-PCR in multiple cell lines (MCF7, ZR75-1 and U-2OS). Regulation of Src Family kinases (SFK) by key players in DNA damage response was assessed by siRNA knockdown along with western blot and qRT-PCR. Dasatinib and siRNA for Fyn and Yes was employed to inhibit SFKs and verify their role in increased migration and invasion in MCF7 cells treated with sublethal doses of Dox.

**Results:**

The results show that sublethal Dox treatment leads to increased migration and invasion in otherwise non-invasive MCF7 breast cancer cells. Mechanistically, these effects were independent of the epithelial mesenchymal transition, were not due to increased cancer stem cell population, and were not observed with other chemotherapies. Instead, sublethal Dox induces expression of multiple SFK—including Fyn, Yes, and Src—partly in a p53 and ATR-dependent manner. These effects were validated in multiple cell lines. Functionally, inhibiting SFKs with Dasatinib and specific downregulation of Fyn suppressed Dox-induced migration and invasion of MCF7 cells.

**Conclusions:**

Overall, this study demonstrates that sublethal doses of Dox activate a pro-invasive, pro-migration program in cancer cells. Furthermore, by identifying SFKs as key mediators of these effects, our results define a potential therapeutic strategy to mitigate local invasion through co-treatment with Dasatinib.

**Supplementary Information:**

The online version contains supplementary material available at 10.1186/s13058-021-01452-5.

## Background

A major challenge for cancer treatment is the balancing of the antitumor activity of chemotherapeutics with attempts to minimize debilitating side effects of the treatment, which can range from mild discomfort to serious life-threatening conditions. Doxorubicin (Dox) is widely used and highly effective for treatment of breast cancer, sarcomas, leukemia, and lymphomas [1, 2]. However, a major side-effect of Dox treatment is a cumulative dose-dependent cardiotoxicity, which often leads to congestive heart failure [1, 2]. As the incidence of cardiotoxicity is strongly correlated with the dose received, studies have explored the maximum threshold of Dox dose that would minimize or prevent cardiac damage in patients [3–5]. However, reports from clinical trials revealed breast cancer patients receiving low dose Dox had reduced disease-free and overall survival compared to patients who received a higher cumulative dose [3, 6]. This raises an important yet relatively unexplored question: How do cancer cells exposed to sublethal doses of Dox behave? Notably, the effect of low dose Dox on cancer cells has not been sufficiently scrutinized.

Dox has multiple cellular effects that can contribute to its activity, with two proposed major mechanisms of action being the generation of reactive oxygen species (ROS) and DNA damage [7, 8]. ROS generation occurs through redox cycling of Dox [9] while in the DNA damage arm, Dox acts by DNA intercalation and covalently binding to Topoisomerase II, forming a ternary complex and generating DNA strand breaks in the process [10]. This turns on a signaling cascade of DNA damage response (DDR) involving Ataxia-telangiectasia-mutated (ATM) and ataxia telangiectasia and Rad3-related (ATR) kinases [11, 12]. These kinases phosphorylate a wide range of substrates [13]—including Chk1 and Chk2 which are thought to be the primary signal transducers in DDR [14]—leading to activation of p53, one of the most well-known and crucial tumor-suppressor proteins [15, 16]. While the roles of p53 in DDR, cell-cycle arrest, and cell death are well established, previously unknown downstream targets of p53 are still being discovered [17, 18]. Among its other cellular effects, Dox can interfere with cellular calcium homeostasis and induce ER stress [19, 20], dysregulate autophagy [21], and induce iron accumulation in the mitochondria [22], and in addition to apoptosis, Dox can induce senescence, fibrosis, and necrosis [23–25]. Interestingly, multiple Dox-resistant cancer cell lines were reported to undergo epithelial to mesenchymal transition (EMT) [26–28]. Furthermore, Dox treatment can increase the cancer stem cell (CSC) population, leading to drug resistance [29, 30].

Src Family kinases (SFKs) including Src, Fyn, and Yes are non-receptor tyrosine kinases that are important players in signaling pathways ranging from cell proliferation and survival to cell adhesion and cytoskeletal reorganization [31–35]. These pathways are tightly controlled in normal healthy cells and are often dysregulated in cancer; thus, SFKs are considered to be potent oncogenes. Indeed, both Src and Fyn have been reported to be key players in tumorigenesis, and upregulation of SFK activity has been linked to increased invasiveness of various cancers [36]. Fyn is upregulated in multiple types of cancers, including breast, prostate, and liver [36–38]. Yes is required for increased cell proliferation and invasion of melanoma cells [39] and was reported to be highly active in colon carcinoma [34, 40]. Fgr and Hck are associated with tumor progression in colorectal cancer [41]. One of the most well-studied functions of Src is its role in cytoskeletal rearrangement in cells. Indeed, cancer cells undergo extensive cytoskeletal reorganization that alters cell adhesion and allows cell migration, two very important steps for cellular invasion and metastasis [42]. Src signaling has also been linked to secretion of matrix metalloproteinase (MMP), enabling breakdown of extracellular matrix (ECM) [43, 44], another key event in cancer cell invasion. Activation of these pathways in tumor cells increases the metastatic potential [44] and leads to poor outcomes. This prompted the development of SFK inhibitors as therapeutics [45]. Dasatinib is a potent SFK inhibitor, and it is used in the clinic for the treatment of Ph^+^ chronic myelogenous leukemia and acute lymphoblastic leukemia in adults and children [46].

While a multitude of pathways contribute to cancer aggressiveness, understanding the underlying mechanism is a crucial first step in managing disease progression. In this study, we investigated the effects of sublethal doses of Dox on multiple cancer cell lines and find that they activate pro-migration and pro-invasion programs involving SFK and MMPs. Although SFK induction was partially dependent on ATR and p53, it was not activated by other DNA-damaging agents. This study offers an insight into the mechanism and poor clinical outcomes associated with sublethal doses of Dox.

## Materials and methods

### Materials

MCF7, MDA-MB-231, and SKBR3 breast carcinoma cells, HeLa cervical adenocarcinoma cells, U-2OS osteosarcoma cells are from American Type Culture Collection (ATCC, Manassas, VA). ZR75-1 breast carcinoma cells are a gift from Dr. Natalia Marchenko (Stony Brook University). RPMI, DMEM, F12/DMEM, and McCoy’s 5A culture medium, fetal bovine serum (FBS), and superscript III reverse transcriptase (RT) are from Life Technologies (Carlsbad, CA). Bio-Rad protein assay was from Bio-Rad (Hercules, CA). Antibodies for total PARP, total Src, phospho-Src (Y416), Yes, p53, ATM, ATR, E-cadherin, n-cadherin, and Vimentin were from Cell Signaling Technology (Danvers, MA). Anti-actin antibody, doxorubicin-HCl (Dox), and Dasatinib were from Sigma (St. Louis, MO). HRP-labeled secondary antibodies were from Santa Cruz Biotechnology (Santa Cruz, CA). Chemiluminescence kit was from Thermo Scientific (Suwanee, GA). Fluorescence-labeled antibodies for CD44 and CD24 were from BD Biosciences (San Jose, CA). Rhodamine phalloidin was from Invitrogen (Carlsbad, CA). Fluoroshield mounting media with DAPI was from Abcam (Cambridge, UK).

### Cell culture and siRNA

MCF-7 and ZR75-1 cells were maintained in RPMI media containing 10% or 15% FBS respectively; MDA-MB-231 and HeLa cells were maintained in DMEM containing 10% FBS. U-2 OS cells were maintained in McCoy’s 5A containing 10% FBS; SKBR3 cells were maintained in F12/DMEM containing 10% FBS. Cell lines were maintained at 37 °C, 5% CO_2_ in a humidified atmosphere and tested for mycoplasma contamination bi-monthly. For experiments, cells were sub-cultured in 60 mm (200 K cells) and 100 mm (500 K cells) dishes with media being changed 1–2 h prior to the start of experiments. For siRNA, cells were transfected using both forward and reverse transfection methods. For forward transfections, cells were seeded in 60 mm (150 K) and transfected the next day with 20 nM negative control (AllStars, Qiagen) or siRNA using oligofectamine or lipofectamine RNAimax (Life Technologies) according to the manufacturer’s protocol (Top2α, Top2β, ATR, ATM, p53, Fyn, Yes siRNA from Life Technologies). For reverse transfection, cells were seeded into media containing siRNA complexes using lipofectamine RNAimax according to the manufacturer’s protocol. In both cases, cells were incubated for 48 h before a media change prior to stimulation.

### Cellular overexpression of p53

The pcDNA-p53-WT plasmid was a generous gift from Dr. Ute Moll (Stony Brook University). MCF7 cells were seeded in 60 mm (1 × 10^6^) and transfected the next day with 500 ng of empty vector (pcDNA) or p53 plasmid using Xtreme gene transfection reagent (Roche, Basel, Switzerland) according to the manufacturer’s protocol for 24 h. Cells were harvested for protein and RNA extractions as described below.

### Protein extraction and immunoblot analysis

To extract cellular protein, cells were scraped in RIPA buffer, lysed by sonication on ice (1 time, 10s) and protein concentration estimated by the Bradford assay. Lysate aliquots were mixed with one-third volume of 4X Laemmli buffer (Bio-Rad) containing 2-mercaptoethanol (Sigma), vortexed for 2–3 s and boiled for 5–10 min. Protein was separated by SDS-PAGE using the Criterion system (Bio-Rad) and immunoblotted as described previously [47].

### Real-time RT-PCR

Total mRNA was extracted using the PureLink RNA Mini Kit (Invitrogen). 0.5–1 μg of RNA was converted to cDNA with the Superscript III Kit for first-strand synthesis (Invitrogen) and samples were diluted to 100 μl with molecular biology-grade dH_2_O. Real-time RT-PCR with Taqman assays were performed on the ABI 7500 real-time system using iTaq mastermix (Bio-Rad). Reactions were run in triplicates in 96-well plates with each reaction containing 10 μl of 2 × iTAQ mastermix, 5 μl of cDNA, 1 μl of Taqman primer probe, and 4 μl of water. The qPCR protocol consisted of 2 min enzyme activation at 95 °C followed by 40 cycles consisting of a 10-s melt at 98 °C, and a 60-s anneal and extension at 60 °C. Ct values were converted to mean normalized expression using the ΔΔCt method using actin as a reference gene. Taqman assays were purchased from Life Technologies. For real-time RT-PCR reactions with sybr green, primers were designed with the Thermo Fisher oligoperfect program (https://www.thermofisher.com/us/en/home/life-science/oligonucleotides-primers-probes-genes/custom-dna-oligos/oligo-design-tools/oligoperfect.html) and validated in silico using the University of California, Santa Cruz (UCSC) in silico-PCR program (http://mgc.ucsc.edu/cgi-bin/hgPcr). Two micrograms total RNA were used for cDNA synthesis using the High Capacity cDNA Reverse Transcription Kit (Applied Biosystems, Thermo Fisher).

### Immunofluorescence and confocal microscopy

This was carried out as previously described with minor modifications [48]. MCF7 cells were grown on poly-D-lysine-coated 35-mm confocal dishes (MatTek Corporation) and treated the following day. Cells were fixed using 4% paraformaldehyde, washed with 1× PBS, and permeabilized with 0.1% Triton X-100. Cells were incubated in rhodamine phalloidin at 1:200 in PBS for 20 min at room temperature and protected from light. Following two washes with PBS, cells were stained with DAPI in mounting media for 10 min according to the manufacturer’s protocol and imaged using a Leica TCS SP8 laser scanning confocal microscope.

### Wound healing assay

Cells were seeded at 90% confluence in 24-well plates in triplicate for each condition. After 24 h, a scratch was introduced across the center of each well using a p10 pipette tip. Cells were washed once with 1× PBS and replaced with fresh media. After 1 h, the wound was imaged using the EVOS XL Core Cell Imaging System and treated immediately after. Images were taken at 24 and 48 h and area of the scratch was measured at 0, 24, and 48 h using the ImageJ wound healing assay.

### Transwell migration assay

Migration assay was performed as previously described with minor modifications [28]. Cells were seeded and treated in 60-mm plates for 24 h or 100-mm plates with siRNA for 48 h followed by stimulation with Dox. Cells were trypsinized and resuspended at 200,000 cells/ml in serum-free media. In total, 500 μl was added to transwell inserts with 8-μm pores (Corning, NY, USA) with 600 μl of complete media added to the lower chamber. Following 24 h in normal culture conditions, excess cells were removed from the upper side of the membrane with cotton swabs and the lower side of the membrane was fixed with 70% ethanol for 10 min. After drying (10–15 min), membranes were stained in 0.2% crystal violet for 5–10 min. Wells were washed with ddH_2_O and air-dried overnight. Migrated cells were imaged using EVOS XL Core Cell Imaging System and quantified using ImageJ multi-point and threshold tool.

### Invasion assay

Cell invasion assay was carried out using the Corning Tumor Invasion System (Corning, NY, USA) following the manufacturer’s protocol with minor modifications as previously described [49]. Briefly, cells were starved for 4 h, resuspended at 200,000 cells/ml, and 500 μl was added to the apical chamber of the preactivated cell invasion plate. Serum-free medium or medium with chemoattractant was added to the lower chamber at a volume of 750 μl and cells were allowed to invade under normal cell culture conditions. Calcein AM dye (Life Technologies) was used to stain invading cells. Fluorescence was quantified using the SpectraMax M5 plate reader (Molecular Devices, Sunnyvale, CA, USA).

### Adhesion assay

Cell-matrix adhesion assay was adapted from Piccolo protocol [50] and reported previously [51]. MCF-7 cells were plated in 100-mm dishes at 1 million cells per each treatment condition in RPMI-high glucose, 10% FBS. Next day, culture media were changed to RMPI-high glucose without FBS, for 16 h, and then treatments were added for the required time. Cells were washed in FBS-free media and detached with 10 mM EDTA in PBS for 20 min and collected with the help of a cell lifter. Cells were collected and centrifuged at 600×*g* for 3 min and resuspended at 10^5^ cells/ml in FBS-free media. Cell culture 12-well plates or 35-mm confocal dishes were previously coated with fibronectin for 16 h at 1μg/ml, and cells were added at 100 K/cells/ml and incubated at 37 °C for 60 min. Culture dishes were washed 3 times with PBS, and cell number was estimated using the MTT assay. Alternatively, cells were fixed with warmed 4% paraformaldehyde in PBS (w/v), permeabilized in 0.1% Triton X-100 (v/v) in PBS, and stained using rhodamine-phalloidin and DAPI. Attached cells were visualized under a laser scanning confocal microscope Leica TCS SP8 at × 20 magnification. Images were processed for automatic cell counting using Python-Bioformats-OpenCV.

### LDH release assay

The LDH assay was carried out using a commercially available kit according to the manufacturer’s protocol. Briefly, at the end of treatment period, 50 μL of medium were placed in duplicates in a 96-well plate. Equal volume of LDH reaction mix was added to each well and covered with aluminum foil to protect from light. The plate was incubated at 37 °C for 30 min and endpoint absorbance was detected at 490 nm and 680 nm wavelength using the SpectraMax plate reader.

### Assessment of viable cell number

Following treatment, cells were washed once with warm PBS. Medium containing 5 mg/ml MTT (3-(4,5-dimethylthiazol-2-yl)-2,5-diphenyltetrazolium Bromide) was added for 30 min at normal cell culture conditions. The insoluble formazan product of MTT was dissolved with DMSO and quantified by measuring its absorbance at 570 nm using the SpectraMax plate reader.

### Analysis of transcriptome by RNAseq

MCF-7 cells were sub-cultured in 100-mm dishes (400 K) and treated with vehicle (Veh) or Dox as described. Total mRNA was extracted using the PureLink RNA Mini Kit (Invitrogen) and > 2 μg of DNase-treated RNA samples were submitted to New York Genome Center for deep sequencing analysis. The transcriptome was analyzed by 2 × 50 paired end sequencing to a depth of 30 million reads using a HiSeq2500. Aligned genes were normalized using Salmon [52] and the resulting lists for each condition were filtered to remove gene counts having a value < 1. Gene counts were compared as a ratio of treated and untreated conditions. List of genes having a ratio value of > 1.95 were input into the Database for Annotation, Visualization and Integrated Discovery (DAVID) for gene ontology analysis (https://david.ncifcrf.gov/tools.jsp).

### Flow cytometry

The assay was carried out as previously described [53] with minor changes. Briefly, cells were sub-cultured in 150-mm dishes (10^6^) and, following treatment, were washed with warm 1× PBS, trypsinized, and centrifuged at 700×*g* for 5 min. Cell pellets were resuspended in PBS at 500,000 cells/100 μl and incubated with FITC-CD24 and PE-CD44 antibodies (1:20 dilution, 4 °C, 30 min). FITC-IgG and PE-IgG were used as negative controls. Following incubation, cells were washed twice with PBS, resuspended in 500 μl PBS, and analyzed using BD Accuri C6 Plus flow cytometer. Data were analyzed using FlowJo software.

### Gelatin zymography assay

Following treatment, cells were rinsed with serum-free media and incubated with fresh serum-free media for 24 h. Conditioned media was harvested on ice, centrifuged for 15 min 2800×*g*, and supernatant concentrated 8-fold using a CentriVap Cold Trap (AL Scientific , Glendale, NY, USA). Concentrated media was mixed with 4× Laemmli buffer (Bio-Rad, Hercules, CA, USA) without boiling or 2-mercaptoethanol. Ten percent SDS-PAGE gels (0.75 mm thick) containing 0.1% gelatin in the resolving gel were prepared. Sample volumes loaded were based on the number of cells counted at the end of zymogen experiments. Following electrophoresis, (Mini-PROTEAN Bio-Rad), gels were removed from their cassettes, rinsed in distilled water, and incubated with 1× Zymogram Renaturation Buffer (Bio-Rad) for 30 min with gentle agitation to remove SDS and renature the proteins. Gels were transferred to a 1× Zymogram Development Buffer (Bio-Rad) for 30 min at room temperature, development buffer was replaced, and gels incubated for 48 h at 37 °C to allow proteolytic digestion of the gelatin substrate. Gels were rinsed with distilled water and stained with Coomassie blue for 30 min. Destaining was carried out with 50% methanol and 10% acetic for 1 h. Zones of gelatin degradation were imaged using an Odyssey CLx Imaging system (LI-COR Biosciences, Lincoln, NE, USA), measured with ImageJ analyzing software, and normalized to the value of vehicle-treated samples.

### Statistical analysis

Data are presented as mean ± SEM. Comparison of two means was performed by unpaired Student’s t test. Comparison of means greater than two was performed by one-way ANOVA. Comparison of experiments with two variables was by 2-way ANOVA with Bonferroni post-test analysis. All analysis was performed using Prism/GraphPad software using a *p* < 0.05 threshold for statistical significance.

## Results

### Sublethal doses of Dox induce pro-invasion/migration phenotype

**Fig. 1:**
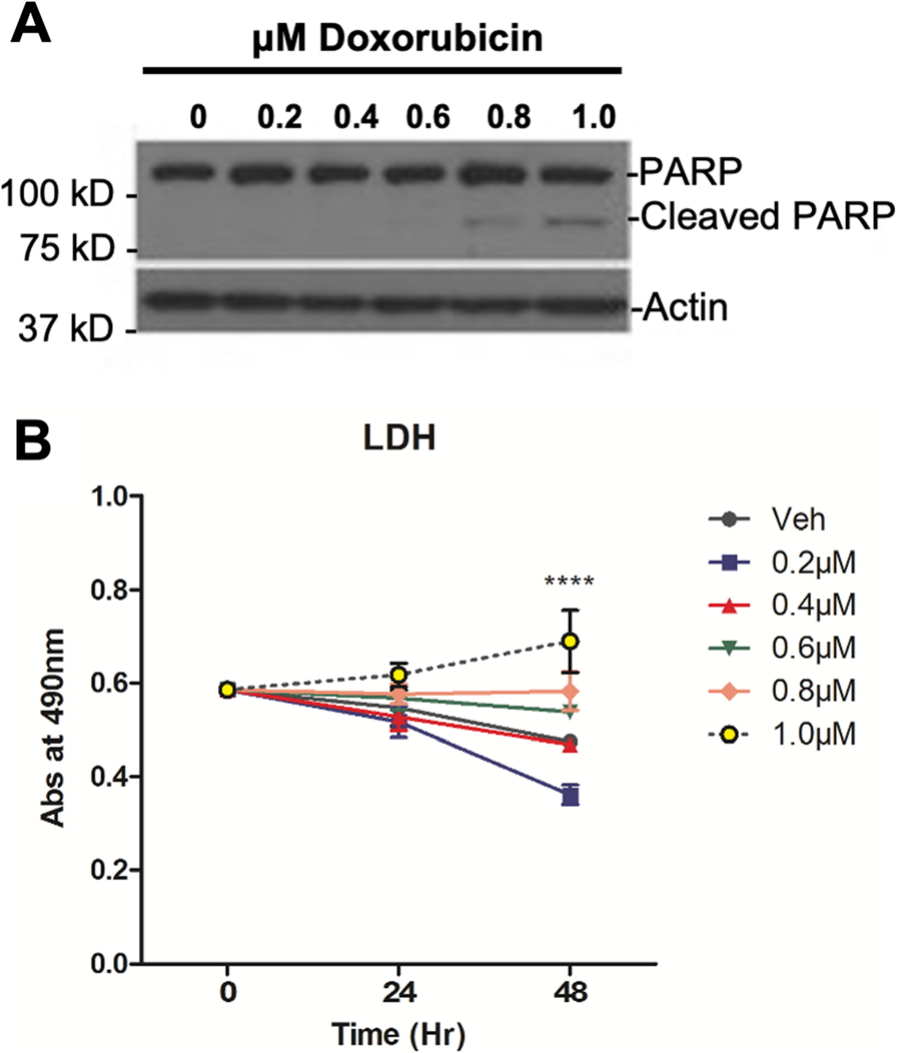
Sublethal doses of doxorubicin in MCF7 cells. Cells were treated with vehicle (DMSO) or Dox for the doses and times shown. **A** Protein was extracted and immunoblotted for total PARP and Actin. **B** LDH release in media was measured as a readout for cytotoxicity (Mean ± SEM, ****p < 0.0001 vs. Veh, *n =* 3)

**Fig. 2:**
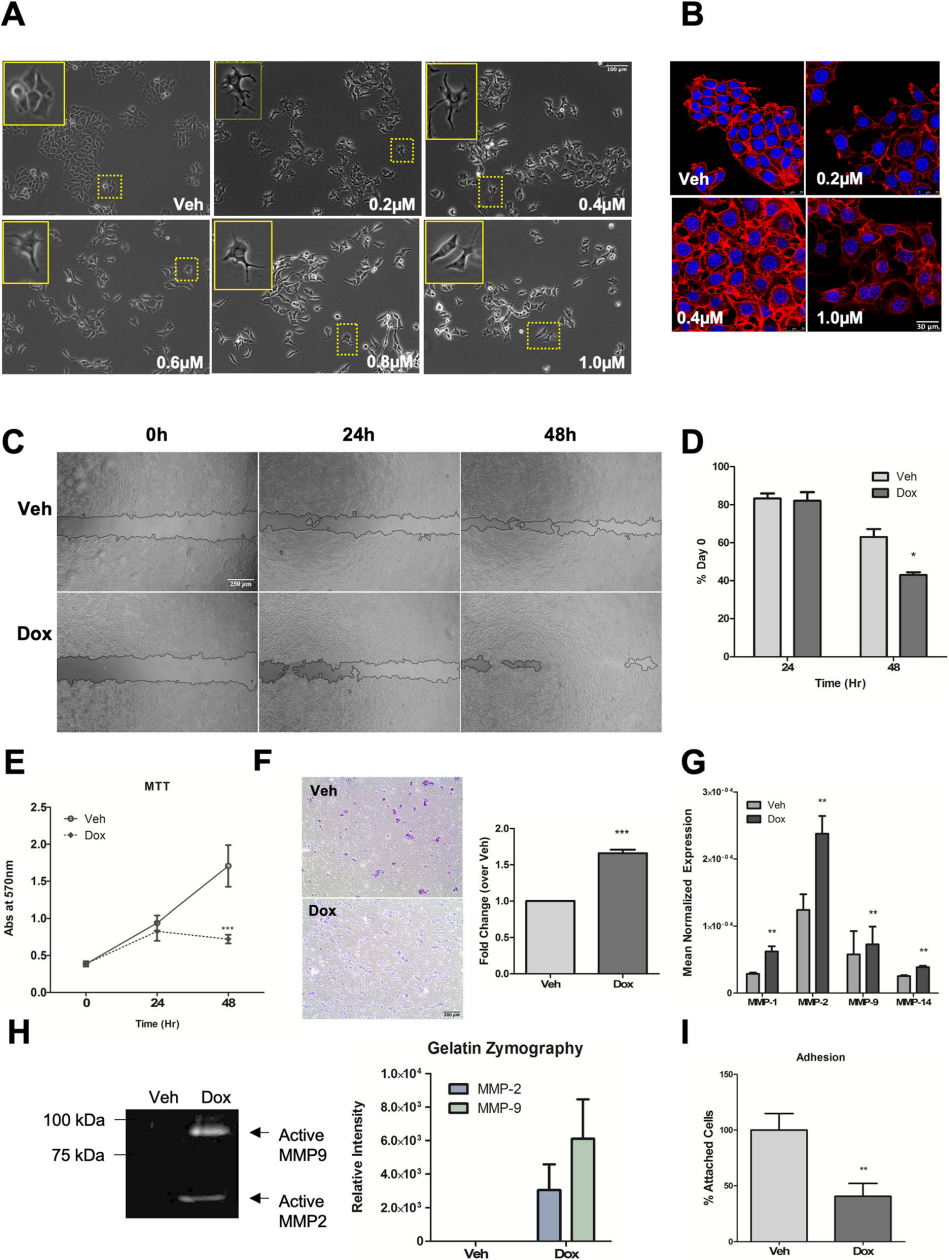
Sublethal doses of Dox induce pro-invasion/migration phenotype. **A** Brightfield images show changes in cell morphology with Dox treatment. Inset shows zoomed in view. **B** Cells were fixed and stained with Rhodamine-Phalloidin to visualize changes in the actin cytoskeleton and DAPI to visualize nuclei. Cells were imaged with a confocal microscope at × 63 magnification. **C** Cells were seeded in 24-well plate, and a scratch was introduced in each well prior to treatment. Random field-of-view of wound healing for each condition was shown at the time points indicated. **D** Quantification of wound area from **C** expressed as % area on day 0 (Mean ± SEM, *p < 0.05 vs. Veh. *n =* 3 in triplicate). **E** Cells were seeded in 6-well plates. At each time point, viable cell number was assessed by MTT assay (Mean ± SEM, ***p < 0.001 vs. Veh, *n =* 3). **F** Cells were treated for 24 h as shown prior to trypsinization and re-seeding in serum-free media into Boyden chambers with 0.8-μm pores, with 10% FBS media as chemoattractant. Migrated cells were stained with crystal violet and random brightfield images were taken with an EVOS microscope. Quantification of migrated cell numbers in **F** from 8 random fields-of-view for each condition. Data are fold change over Veh. (Mean ± SEM, ***p < 0.0005 vs. Veh *n =* 3). **G** qRT-PCR analysis of MMP-1, MMP-2, MMP-9, and MMP-14 was performed with actin as reference gene. Data are shown as mean normalized expression (Mean ± SEM, **p < 0.01 vs. veh, *n* = 3). **H** Cells were serum starved for 24 h post-treatment. Conditioned media was collected, and gelatin zymography assay was done to assess MMP-2 and MMP-9 activity. Picture shown is representative of *n* = 3. Quantification of gelatin zymography; relative intensity of bands in gel plotted (*n* = 3). **I** Cells were seeded in 100-mm dishes and serum starved for 16 h prior to treatment, followed by re-seeding in fibronectin-coated 12-well plates. After 1 h, the number of attached cells following multiple washes with PBS was estimated with MTT assay. Expressed as % attached cells of veh (Mean ± SEM, **p < 0.01 vs veh, *n* = 4). All treatments with Dox are at 0.4 μM unless otherwise noted

To establish appropriate sublethal doses of Dox and to assess the cellular effect of sublethal Dox treatment, MCF7 cells were treated with Dox ranging from 0.2 to 1.0 μM for 24 h. Immunoblot analysis showed PARP cleavage did not occur at Dox doses of 0.2 μM, 0.4 μM, and 0.6 μM but was observed at 0.8 μM and 1.0 μM Dox, indicating initiation of apoptosis at these higher concentrations (Fig. 1A). Consistent with this, lactate dehydrogenase (LDH) release was not observed at doses up to 0.8 μM Dox, but significant release of LDH occurred at the apoptotic dose of 1.0 μM, indicating loss of membrane integrity at this dose (Fig. 1B). Based on these results, doses of 0.6 μM or less were denoted as sublethal. Notably, cells treated with sublethal doses of Dox displayed changes in cell shape (Fig. 2A), with protrusions extending from the cell body following treatment that gave the cells a stellate shape. This filopodia-like morphology was most prominent in at 0.2 μM and 0.4 μM Dox, and while protrusions were present at 0.6 μM, 0.8 μM, and 1.0 μM Dox, the cells appeared more elongated. Phalloidin staining highlighted that the change in cell shape indeed affected the actin cytoskeleton. Untreated cells were polygonal in shape while Dox-treated cells appeared more multipolar (Fig. 2B). As changes in cell shape at sublethal doses of Dox suggested a more invasive phenotype, we sought to evaluate this experimentally. Accordingly, a wound healing assay was performed. Following introduction of a scratch, cells were treated with vehicle or 0.4 μM Dox, and wound closure was monitored. As can be seen, Dox-treated cells closed the wound significantly more compared to vehicle-treated cells (Fig. 2C, D). To rule out effects on cell growth as a reason for wound closure, viable cell numbers of vehicle- and Dox-treated cells were assessed (Fig. 2E). Strikingly, vehicle-treated cells retained normal cell growth while a growth arrest was observed in Dox-treated cells. Thus, the wound closure observed is not a result of enhanced cell growth (Fig. 2E). Consolidating these data, cells treated with sublethal doses of Dox showed significantly increased motility in a transwell migration assay compared to vehicle-treated cells (Fig. 2F). Consistent with induction of a more invasive phenotype, there was a significant increase in expression of MMPs, most evident with MMP-1 and MMP-2 increasing by 2.17-fold and 1.92-fold respectively (Fig. 2G). This was further reflected in the increased secreted activity of MMP-2 and MMP-9 following sublethal Dox treatment with little to no activity in untreated cells (Fig. 2H). Finally, cells treated with sublethal Dox showed a significant reduction in the ability of cells to attach on fibronectin (Fig. 2I). Of note, while we also observed a reduction of cell growth following Dox treatment, removal of Dox allowed cells to resume growth (Supplemental Fig. 1). Taken together, these results show that sublethal Dox treatment induces a pro-migratory and pro-invasive program in non-invasive MCF7 cells.

### Sublethal Dox treatment induces migration and invasion without inducing an EMT or increasing the cancer stem cell population

**Fig. 3:**
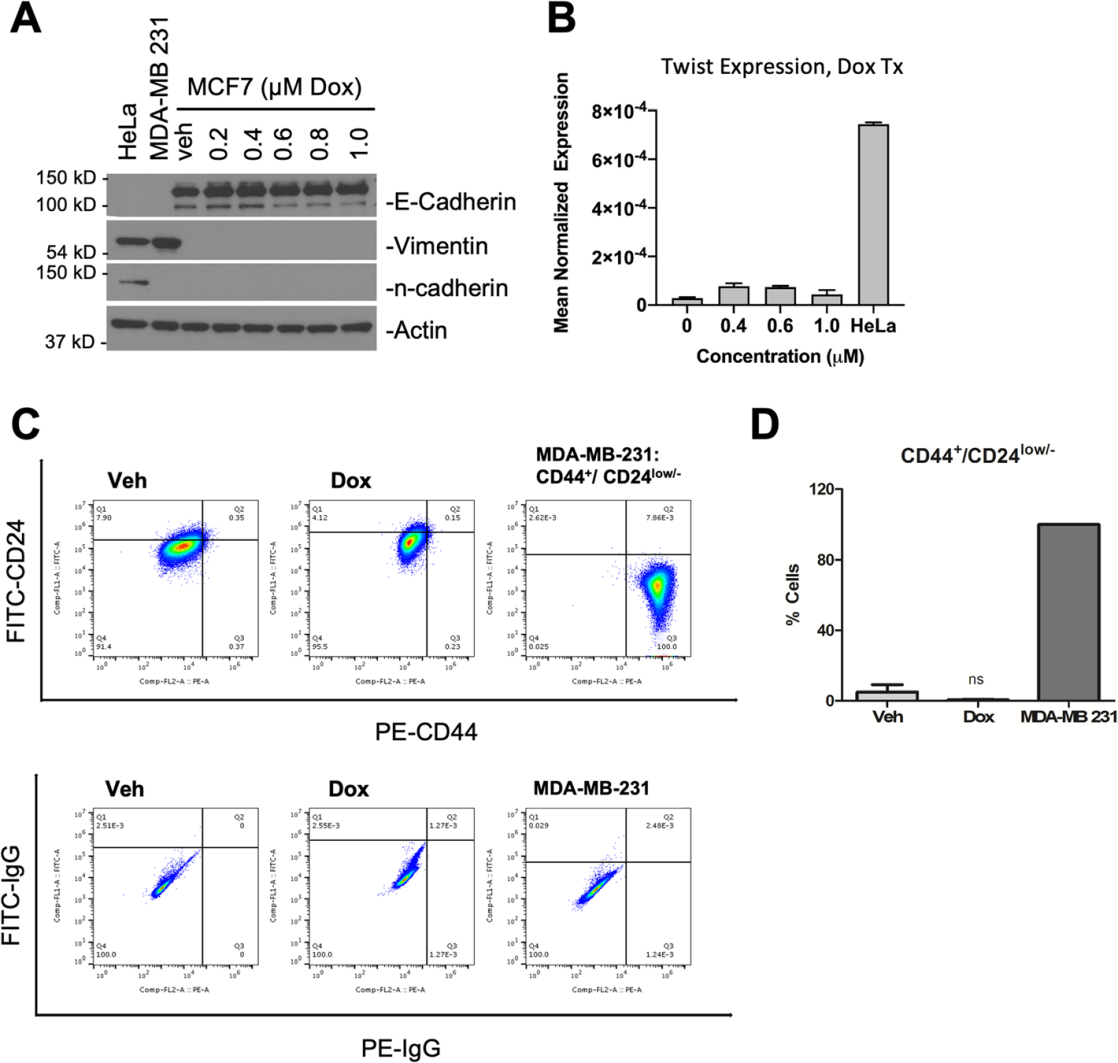
Sublethal Dox treatment induces migration without inducing an EMT or increasing the CSC population. MCF7 cells were treated with vehicle (DMSO) or 0.4 μM Dox as shown. **A** Protein was extracted and immunoblotted for E-cadherin, Vimentin, N-cadherin, and Actin. HeLa and MDA-MB-231 cell lysates were used as negative controls for E-cadherin expression and positive controls for Vimentin and N-cadherin expression. **B** Twist1 expression was analyzed by qRT-PCR using actin as reference gene. HeLa cells were used as a positive control. Data is expressed as mean normalized expression ± SEM (*n* = 3). **C** For analysis of the CSC population, cells were stained with FITC-CD24 and PE-CD44 antibodies and analyzed by flow cytometry. MDA-MB-231 cells were used as positive control. **D** Quantification of CD44^+^/CD24^low/−^ population in veh/Dox-treated MCF7 cells with MDA-MB-231 cells as positive control (*n* = 4)

The epithelial-mesenchymal transition (EMT) is a well-established promoter of cell invasion and can lead to the generation of cells with cancer stem cell (CSC) characteristics. In the EMT, cells lose epithelial characteristics and transition to display more mesenchymal features resulting in increased motility [54]. To determine if EMT is relevant in the effects of Dox here, EMT markers were assessed by immunoblot (Fig. 3A). Interestingly, cells treated with sublethal Dox doses retained expression of the epithelial marker E-cadherin and did not express the mesenchymal markers Vimentin or N-cadherin. Consistent with this, sublethal Dox treatment did not significantly increase the expression of Twist (Fig. 3B), a key transcription factor for EMT. Consistent with these findings, there was no appreciable change in the CSC population (CD44^+^/CD24^low/−^) between vehicle-treated and Dox-treated cells (Fig. 3C, D), indicating sublethal Dox treatment did not result in an enrichment of CSCs. Taken together, these results showed that sublethal Dox treatment induces migration and invasion independently of EMT or increasing CSC population.

### Sublethal Dox treatment results in enrichment of genes regulating cell migration and cell-cell adhesion

**Fig. 4:**
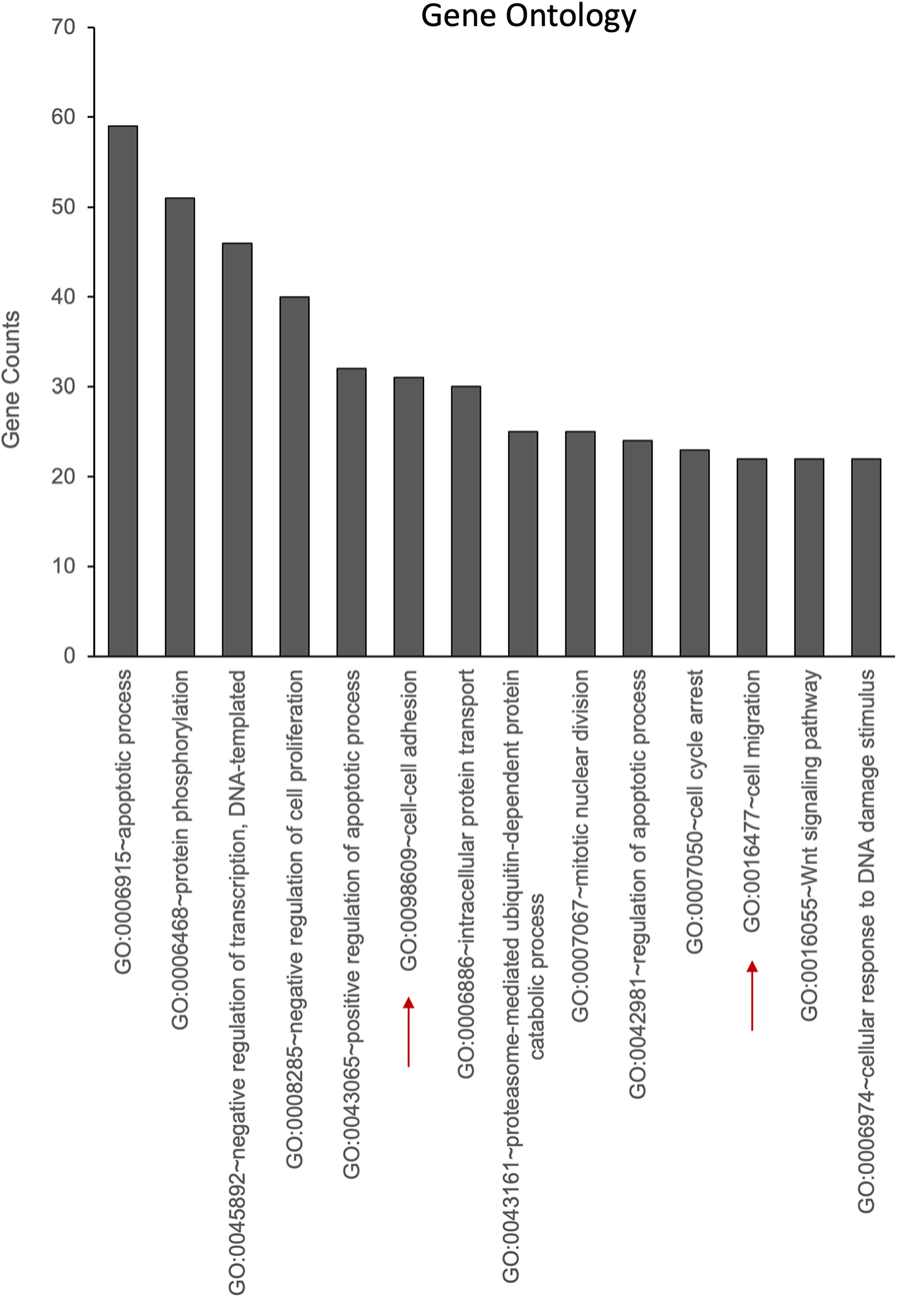
Sublethal Dox treatment induces expression of genes regulating cell-cell adhesion and cell migration. MCF7 cells were treated with vehicle (DMSO) or 0.4 μM Dox as shown. RNA was extracted and subject to RNAseq analysis. Pathways enriched after Dox treatment were plotted by gene counts

**Fig. 5:**
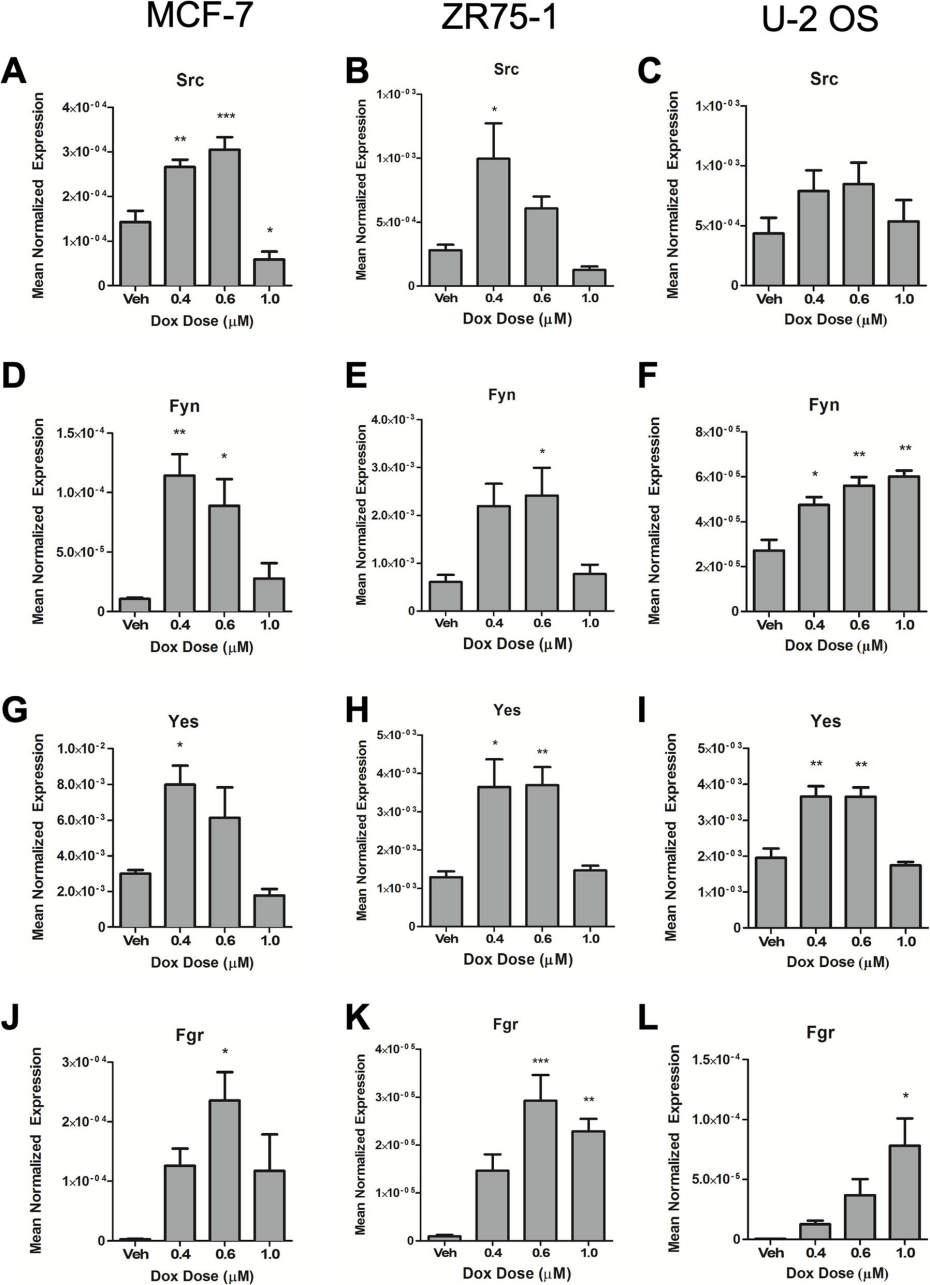
Validation of Src family kinase induction by Dox treatment. qRT-PCR analysis of Src, Fyn, Yes, and Fgr was performed using actin as reference gene in **A**, **D**, **G**, **J** MCF7, **B**, **E**, **H**, **K** ZR75-1, and **C**, **F**, **I**, **L** U-2OS cells. Data is presented as mean normalized expression (Mean ± SEM, *p < 0.05, **p < 0.01, ***p < 0.005 vs. vehicle, *n =* 4 in MCF7, *n =* 5 in ZR75-1, and *n =* 3 in U-2OS)

To understand how sublethal Dox may affect the biology of the cell, we performed a discovery RNAseq analysis on MCF7 cells in order to define changes in gene expression following sublethal Dox treatment. Consistent with the observed phenotypes, data analysis showed that genes regulating cell adhesion and cellular migration were enriched in cells treated with sublethal doses of Dox (Fig. 4). Interestingly, multiple Src Family Kinases (SFK) were induced by Dox in MCF7 cells (Table 1). To confirm SFK upregulation was indeed at the mRNA level, Src expression was assessed by real-time PCR. Results in MCF7 cells showed significant increases in Src mRNA at 0.4 μM (1.90-fold) and 0.6 μM Dox (2.10-fold) yet at 1 μM Dox—an apoptotic Dox dose—Src was not induced (Fig. 5A). Beyond Src, there are currently nine members of the SFK including Fyn, Yes, Blk, Yrk, Fgr, Hck, Lck, and Lyn. Of these, Src, Fyn, and Yes are expressed in a wide range of tissues [33] and have been shown to promote cell migration/invasion [35, 40, 55]. Extending our analysis revealed marked induction of Fyn by Dox, peaking at 0.4 μM (10.7-fold) and markedly lower at apoptotic doses of 1 μM (2.6-fold) (Fig. 5D). Similarly, Yes expression was induced by Dox with a peak at 0.4 μM (2.66-fold) with no induction at higher doses (Fig. 5G). Finally, Fgr was induced at 86.5-fold at 0.6 μM (Fig. 5J). To consolidate and extend these results, we utilized ZR75-1, MDA-MB-231, and SKBR3 breast carcinoma and U-2OS osteosarcoma cells (Fig. 5, Supp. Fig. 2). Real-time analysis showed that sublethal Dox treatment of ZR75-1 cells leads to similar induction of Src (3.54-fold at 0.4 μM)(Fig. 5B), Fyn (3.62-fold)(Fig. 5E), Yes (2.82-fold)(Fig. 5H), and Fgr (15.4-fold) (Fig. 5K). Similarly, Dox treatment of U-2OS cells showed increased Src (1.81-fold at 0.4 μM)(Fig. 5C), Fyn (1.75-fold), (Fig. 5F) Yes (1.87-fold)(Fig. 5I), and Fgr (39-fold)(Fig. 5L). To evaluate if the increase in mRNA was reflected at the protein level, Dox effects on Src and Yes in MCF7, ZR75-1, and U-2 OS cells were assessed by immunoblotting (Supp. Fig. 4A-C). Results showed that Dox treatment showed a marked increase of Src at 0.4 μM to 1.0 μM in MCF7 cells (Supp. Fig. 4A). In ZR75-1 cell, 0.4 μM Dox showed an increase in both Src and Yes levels (Supp. Fig. 4B). Similar results were seen in U-2 OS cells with increased levels of Src at 0.6 μM and Yes at 0.4 μM (Supp. Fig. 4C). Importantly, in all cell lines, increased levels of Src were accompanied by increased levels of phospho-Src (Tyr-416), indicating that Dox is inducing the active form of the protein (Supp. Fig. 4).

**Table 1:** Members of Src Family Kinases are upregulated following Dox treatment

Gene Symbol	Veh (normalized counts)	Dox (normalized counts)	Fold-change (Dox/Veh)
SRC	0.87	3.94	4.52
FYN	1.1	4.27	3.9
YES1	4483.77	9752.9	2.18
FRK	115.09	88.2	0.77
BLK	2.03	1.09	0.54
FGR	-	-	-
HCK	1.38	3.35	2.42
LCK	7.35	0.42	0.06
LYN	2.7	-	-

### Dox induction of Fyn and Yes is partially dependent on p53

**Fig. 6:**
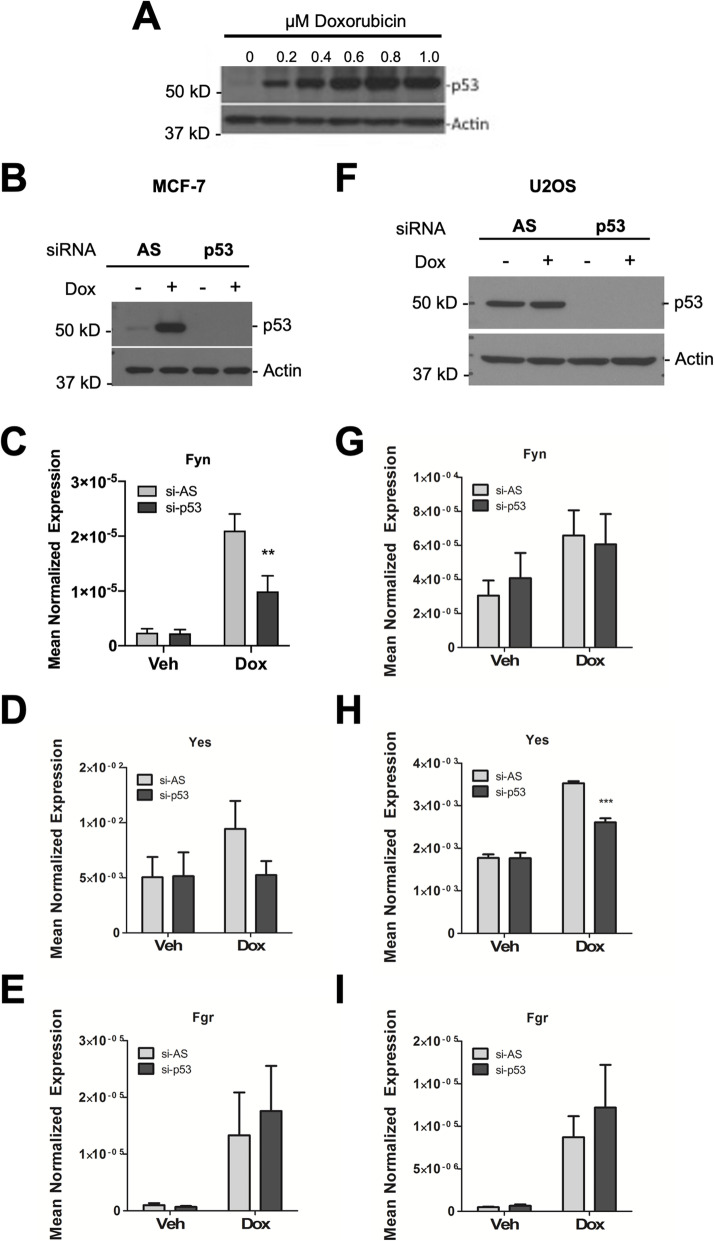
Dox induction of Fyn and Yes is dependent on p53. MCF7 and U-2OS cells were treated with siRNA, vehicle (DMSO) and 0.4 μM Dox as shown. (**A**) Protein was extracted and immunoblotted for p53 and Actin in MCF7 cells. (**B-I**) Allstars negative control (AS) or p53-siRNA-treated cells were analyzed for (**B,F**) p53 knockdown by immunoblot and (**C,G**) Fyn (**D,H**) Yes (**E,I**) Fgr expression by qRT-PCR analysis in MCF7 and U-2OS cells (Mean ± SEM, **p < 0.01 vs. si-AS, *n* = 4 in MCF7, *n* = 3 in U-2OS)

Dox binds to DNA and Topoisomerase II (Top2) and generates DNA strand breaks which leads to a cascade of events involving p53 ref. [56]. Indeed, in MCF7 cells, there was a marked dose-dependent increase in the levels of p53 in cells treated with Dox **(**Fig. 6A). To understand the mechanism of action, we sought to explore whether p53 played a role in the induction of Fyn by Dox using an siRNA approach. Results showed a partial dependence of Fyn induction on p53 in MCF7 cells (Fig. 6B, C) but this was not seen for Yes or Fgr (Fig. 6D, E). Similarly, Fyn induction was partially dependent on p53 in ZR75-1 cells (Supp. Fig. 5A-C). In contrast, in U-2OS cells, Yes induction was dependent on p53 expression (Fig. 6F–I). Mutations of p53 that lead to a disruption of its transcriptional activity is a common event in many cancers. Accordingly, to determine if Dox had similar effects in the context of p53 mutations, we extended our studies into MDA-MB-231 (basal) and SKBR3 (HER2+) cells (Supp. Fig. 2). Both cell lines harbor p53 mutation and, importantly, represent distinct subtypes of breast cancer than the luminal, ER-positive MCF7 and ZR75-1 cells. Results in MDA-MB-231 cells showed that Dox did induce SFKs, increasing Src (1.98-fold at 0.4 μM Dox), Fyn (2.17-fold at 0.6 μM Dox), and Yes (1.75-fold at 0.6 μM Dox) although this was considerably less than seen in MCF7 and ZR75-1 cells. Interestingly, Dox treatment of SKBR3 cells had no effect on Src, Fyn, and Yes within the range of 0.4–1.0 μM. Finally, to determine if p53 is sufficient to drive SFK levels, the effects of transient p53 overexpression on the mRNA levels of SFKs were assessed. As can be seen, we achieved a modest overexpression of p53 at the protein level. Importantly, this was associated with a modest but statistically significant increase in Fyn expression but not in Src and Yes (Supp. Fig. 5D-G). Taken together, these results suggest that Dox effects on SFKs are at least partially p53 dependent.

### Induction of Src family kinases and migration is not a general response of DNA-damaging agents and chemotherapies

**Fig. 7:**
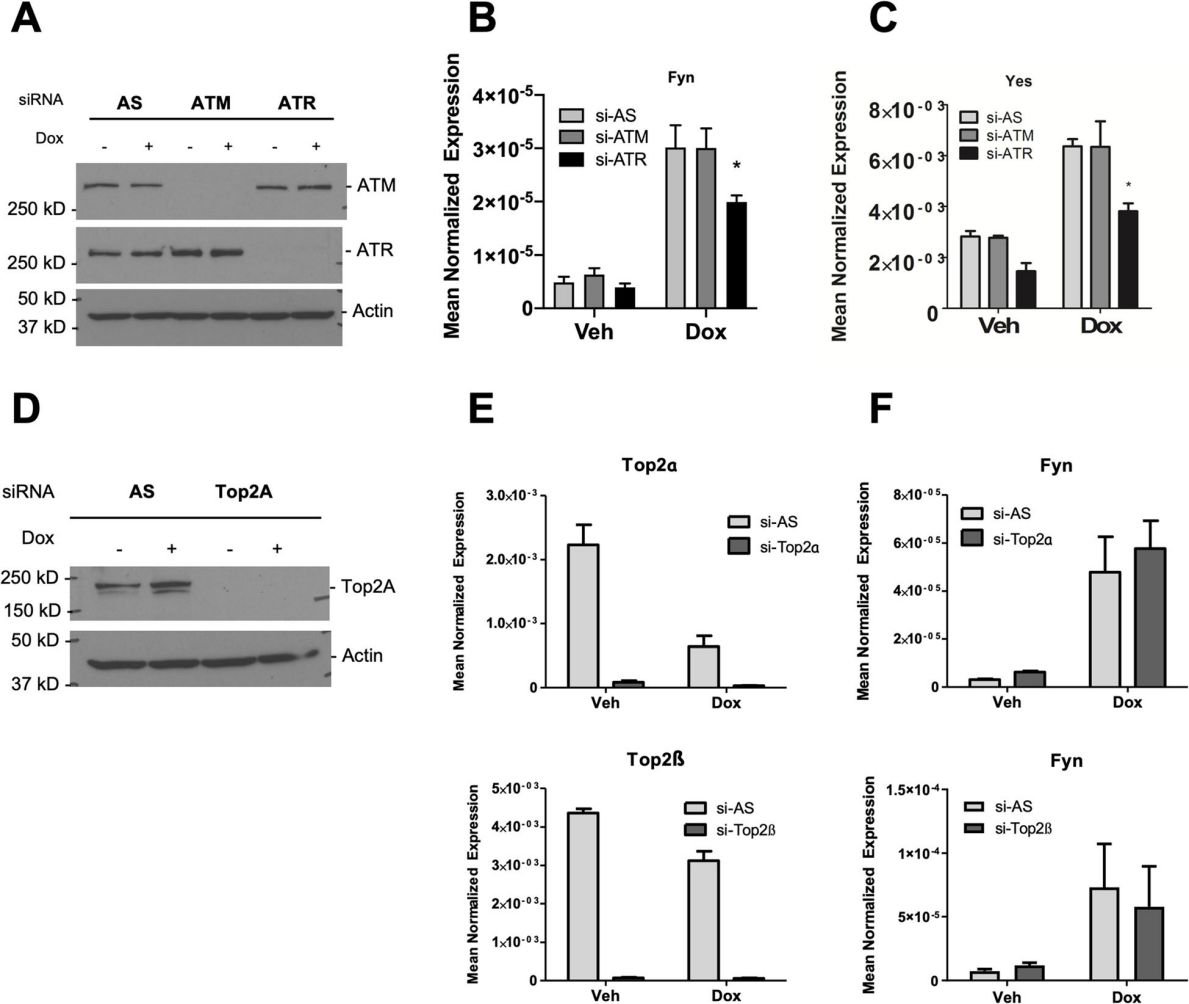
Dox induction of Fyn is dependent on ATR but not Topoisomerase II. MCF7 cells were treated with siRNA, vehicle (DMSO), and 0.4 μM Dox as shown. **A–C** Cells treated with AS negative control, ATM, or ATR siRNA were analyzed for **A** protein by immunoblot for ATM, ATR, and Actin. **B** Fyn and **C** Yes expression by qRT-PCR analysis (Mean ± SEM, *p < 0.05, *n =* 5). **D–F** Cells treated with AS negative control, Top2A-siRNA, or Top2B-siRNA were analyzed for **D** Top2A knockdown by immunoblot and **E** Top2A and Top2B; and **F** Fyn by qRT-PCR. For all analyses, actin was used as a reference gene. Data is presented as mean normalized expression (Mean ± SEM, *n =* 3)

**Fig. 8:**
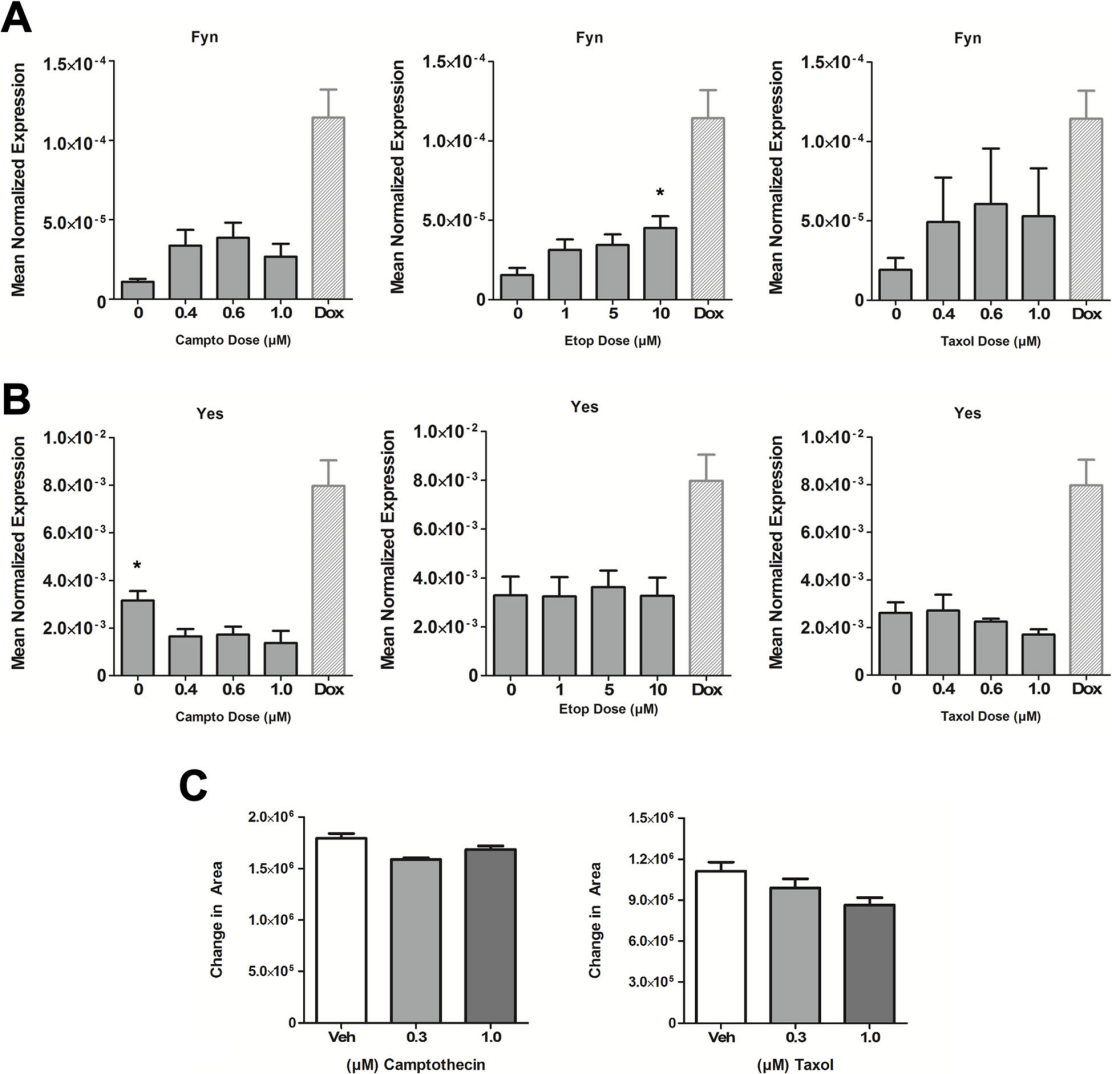
Induction of Src family kinases and migration is not a general response to chemotherapies. MCF7 cells were treated with vehicle (DMSO) or doses of Camptothecin, Etoposide, or Taxol as shown. **A** Fyn and **B** Yes expression were analyzed by qRT-PCR with actin as a reference gene. Results on Dox from Fig. 5 shown to allow comparison. Data are shown as mean normalized expression (Mean ± SEM, *p < 0.05 vs veh, *n =* 4; results from Dox induced are not included in the statistical calculations). **C** Cells were seeded in 24-well plate, and a scratch was introduced in each well prior to treatment with Camptothecin or Taxol. Change in area of wound is shown

The involvement of p53 in SFK induction led us to speculate that this could be linked to the DNA damage response (DDR). To begin to explore this, the role of the upstream DDR kinases ATM and ATR in SFK induction was assessed in MCF7 cells (Fig. 7A–C). Here, knockdown of ATR but not ATM showed a modest but statistically significant effect on both Fyn and Yes induction (Fig. 7B, C). The interaction of Dox with Top2α and Top2β is an important part of how it induces DNA double-strand breaks. However, siRNA knockdown of either Top2α or Top2β had no significant effects on Fyn induction (Fig. 7D–F). The connection of SFK induction to the ATR-p53 axis prompted us to assess if other DNA-damaging agents had the same effects. Interestingly, MCF7 cells treated with Camptothecin and Etoposide did not show increases in either Fyn (Fig. 8A) or Yes (Fig. 8B). Similarly, treatment with Taxol, a cytoskeletal targeting drug, also did not change expression levels of Fyn and Yes mRNA (Fig. 8A, B). Consistent with these findings, treatment with Camptothecin or Taxol did not induce migration of MCF7 cells in the wound healing assay (Fig. 8C). Collectively, while these agents are all known to induce the DDR and p53, these results suggest a Dox-specific mechanism is required for induction of SFKs in MCF7 cells.

### Inhibition of Src family kinases prevents Dox-induced invasion/migration

**Fig. 9:**
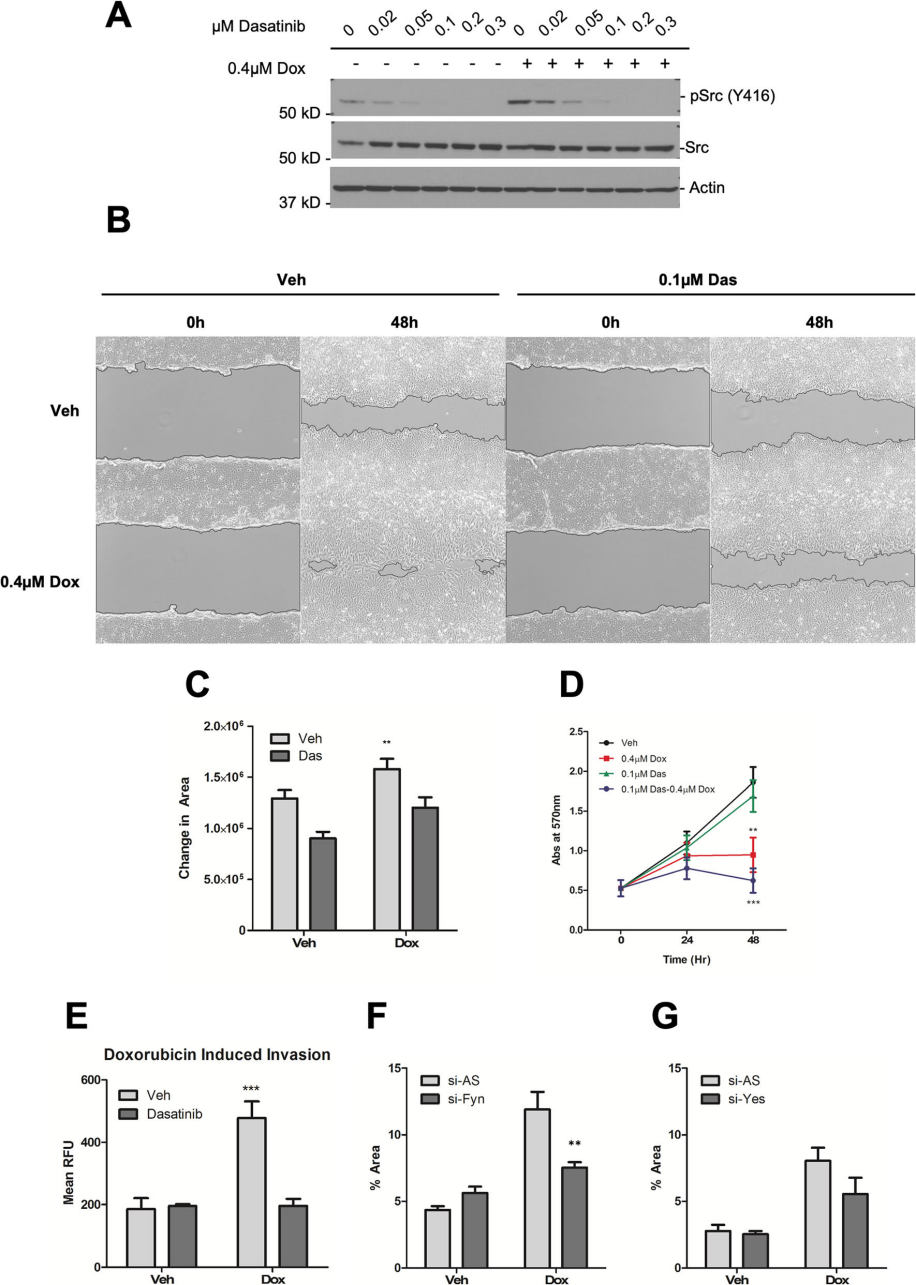
Inhibition of Src family kinases prevents Dox-induced invasion/migration. MCF7 cells were treated with vehicle (DMSO), Dox (0.4 μM), and Dasatinib as shown. **A** Protein was extracted and immunoblotted for phospho-Src (Y416), total Src and actin. **B, C** Cells were seeded in 24-well plate, and a scratch was introduced in each well prior to treatment. **B** Random field-of-view of wound healing for each condition shown. **C** Quantification of area of wound (Mean ± SEM, **p < 0.005 vs. veh, *n =* 3 in triplicate). **D** Cells in 6-well trays were treated as shown and viable cell number assessed by MTT assay at the time points indicated (Mean ± SEM, **p < 0.01 ***p < 0.001 vs. veh, *n =* 3). **E** Cells were treated for 24 h as shown prior to trypsinization and re-seeding into Boyden chambers with 0.8-μm pores with Matrigel overlay. 10% FBS media was used as chemoattractant. Migrated cells were stained with calcein AM dye and detected by fluorescence. Data shown as mean relative fluorescent unit (Mean ± SEM, ***p < 0.001 vs. veh/Dasatinib, *n =* 3 in triplicate). **F, G** MCF7 cells were treated with siRNA, vehicle (DMSO), and 0.4 μM Dox as shown. Cells treated with AS negative control, Fyn, or Yes siRNA, were trypsinized and re-seeded in serum-free media into Boyden chambers with 0.8-μm pores, with 10% FBS media as chemoattractant. Migrated cells were stained with crystal violet and random brightfield images were taken with EVOS microscope. Migrated cells were quantified from 5 random fields-of-view for **F** Fyn and **G** Yes (Mean ± SEM, **p < 0.01 vs. si-AS, *n* = 3 in duplicates). All treatments are at 0.4 μM Dox and 0.1 μM Dasatinib unless otherwise noted

The increase in SFK expression following sublethal dose of Dox prompted us to assess its role in the migratory phenotype. For this, and as multiple SFKs were induced on Dox treatment, a pharmacological approach with the SFK inhibitor Dasatinib was used. Co-treatment with 0.1 μM Dasatinib and 0.4 μM Dox showed inhibition of SFK phosphorylation on Tyr-416 (Fig. 9A). Results from wound healing assays showed Dasatinib co-treatment significantly attenuated the migration of Dox-treated cells (Fig. 9B, C). As before, this was not a consequence of alterations in cell growth (Fig. 9D). Furthermore, Dasatinib co-treatment also significantly mitigated the invasion of Dox-treated cells through Matrigel (Fig. 9E). To further assess the effect of specific SFKs on migration of Dox-treated cells, a genetic approach was employed (Supp. Fig. 6A-C, Fig. 9F,G). As can be seen, knockdown of Fyn showed significant reduction of cell migration through the transwell (Fig. 9F). Migration was also reduced by knockdown of Yes, albeit not significantly (Fig. 9G). Taken together, these results suggest a role of SFKs in the Dox-induced invasion/migration in MCF7 cells and identify Fyn as a major contributor for this effect.

## Discussion

Despite its efficacy as a chemotherapeutic, the clinical utility of Dox—as with other chemotherapies—is hampered by a number of side effects. Although reducing Dox doses has been explored as a strategy to minimize such toxicities, the effects of such doses on cancer cells are relatively unexplored. In this study, we have investigated the effects of sublethal Dox treatment in non-invasive MCF7 cells and other breast cancer cells and find that it leads to increased migration and invasion. Mechanistically, these effects were independent of the EMT, were not due to increased CSC population and were not observed with other chemotherapies. Instead, sublethal Dox led to transcriptional induction of multiple SFK isoforms, partly in a p53 and ATR-dependent manner, and increased induction and secretion of MMP isoforms. Functionally, inhibiting SFKs with Dasatinib inhibited migration and invasion of MCF7 cells resulting from sublethal Dox treatment. Genetic knockdown approaches identified Fyn as a significant contributor to the Dox-induced effect. This study demonstrates that sublethal doses of Dox activates SFK signaling as a key pathway in cancer invasion, which could increase the risk of recurrence in patients receiving suboptimal dose of Dox treatment.

The major finding of our study is the transcriptional activation of a pro-migration, pro-invasion program by sublethal Dox treatment of a non-invasive luminal breast cancer (BC) cell line via SFK signaling. This was initially suggested by phenotypic observations and confirmed by functional assays demonstrating enhanced wound healing and transwell migration induced by Dox. This was further supported with our exploratory RNAseq analysis that showed an enrichment of genes involved in adhesion and migration in cells treated with sublethal Dox. Not surprisingly, genes regulating apoptotic and cell-cycle regulation pathways were also enriched. Doxorubicin is a potent DNA-damaging agent and even sublethal doses of Dox treatment result in DNA intercalation (data not shown) and activate the DDR (e.g., induction of p53). Although lower Dox levels can lead to fewer DNA strand breaks, it nonetheless could activate transcription of genes in anti-growth/pro-death pathways without full execution of apoptosis. Notably, while Dox was previously shown to increase migration and invasion in BC cells [57–59] and osteosarcoma cells [60], this was in the context of more aggressive and already invasive cells such as MDA-MB-231, 4 T1, and U-2OS. In contrast, MCF7 is a “poorly aggressive and non-invasive” cell line, has low metastatic potential [61], and is often used as a negative control in migration/invasion studies [62]. Mechanistically, the pro-invasive effects of Dox are independent of the EMT pathway, a major driver of cancer invasion and previously shown to enhance invasion of MCF7 cells [63–66]. Consistent with the absence of EMT, there was no enrichment of the CSC population in Dox-treated MCF7 cells. This contrasts with prior studies where Dox was reported to induce EMT in BC cells [63]. The reasons for these discrepancies are quite likely related to variation in stimulation time—24 h in this study compared to 48 h in previous reports [65]. Thus, while it is possible that an EMT phenotype may manifest in MCF7 cells at later time points, the temporal differences suggest that an EMT phenotype is not a pre-requisite for Dox induction of invasion. This is in accord with studies reporting that EMT is not mandatory for cells to be invasive [67]. Furthermore, attempts to find correlation between EMT markers and patient prognosis for different types of cancer have been deemed unreliable [68]. The findings in this study further support the idea that tumor cells may be prone to invade/migrate despite the absence of tell-tale signs of EMT. Therefore, patients receiving Dox may be at a higher risk of disease recurrence from metastasis and should be monitored with vigilance.

SFKs are well-known regulators of cell motility, and upregulation of SFK signaling is a common occurrence in cancer [31]. The effects of sublethal Dox on multiple SFK members (Fyn, Yes, Fgr, and Src) pinpointed them as major regulators of Dox-induced migration/invasion. Consistent with this, the profiles of SFK inductions broadly coincided with increases in cell migration and invasion, being higher at sublethal Dox and lower at more apoptotic doses. This was verified functionally with knockdown of Fyn, as well as the broad SFK inhibitor Dasatinib, which effectively blocked the Dox-induced migration/invasion. A previous study demonstrated induction of Src phosphorylation by Dox in MDA-MB-231 cells [69, 70] and in HCT-116 colon cancer cells [69, 70]; however, these studies did not explore effects of Dox on the SFKs, the role of SFKs in Dox-induced migration, or the transcriptional upregulation of SFK by Dox. To the best of our knowledge, the current study is the first to show that sublethal Dox treatment increases multiple SFK at the mRNA level. The cellular stress exerted by Dox treatment is known to induce changes in transcription in yeast and mammalian cells [7, 71, 72]. Future studies are required to understand the mechanisms that drive these transcriptional changes.

Combining Dasatinib with Dox has been shown to synergistically inhibit growth as well as migration and invasion of MDA-MB-231 cells [73] and had a synergistic anti-proliferative effect in a highly tumorigenic ovarian cancer cell line that had high Src levels [74]. In a Dox-resistant sarcoma cell line, combination treatment of Dox with Dasatinib was shown to decrease cell viability [75], and Dasatinib increased therapeutic efficacy of Dox in Dox-resistant hepatocellular carcinoma cells [76]. However, all these studies probed the efficacy of combination treatment in highly aggressive cells utilizing lethal doses of Dox treatment. Here, we have explored the activation of an invasive pathway in non-invasive MCF7 cells using sublethal Dox doses. It has been suggested that there exists a “dichotomy between cell cycle and cell invasion” such that cancer cells undergo cell-cycle arrest in order to engage in invasive behavior, partly contributed by the hijacking of cell-cycle machinery for invasive biology [77]. Indeed, cells treated with sublethal Dox underwent growth arrest while migrating in wound healing assay. We had previously shown that sublethal Dox induces cell-cycle arrest of MCF7 partly in S phase [17]. Other studies have shown effects of doxorubicin on the G2/M and/or G1/S phases of the cell cycle in bladder cancer cells [78] and G0/G1 and G2 in colon cancer cells [79]. However, it is important to note that cells resumed growth following removal of Dox, suggesting that growth arrest induced by sublethal Dox is reversible. A minor limitation of our study is that we did not assess other targets of Dasatinib that may also be involved—for example, Eph2A, c-kit, and PDGFRβ. Nevertheless, as Dox increased multiple SFK members, this suggests that there could be potential redundancy and overlap among isoforms which would make such an analysis less clear cut. Moreover, knockdown of Fyn specifically mimicked the action of Dasatinib. Critically, the use of Dasatinib, which is an FDA-approved SFK inhibitor that is already in clinical use, enhances the translational potential of these findings.

One of the key events in invasion by cancer cells is the breakdown and remodeling of ECM by matrix metalloproteinases [80]. It is well established that increased expression of MMP-2 and MMP-9 have been evaluated as prognostic marker in BC and prostate cancer [81, 82]. In addition, MMP-9 overexpression has also been correlated with poor prognosis in colon, gastric, lung, and pancreatic cancers [83]. Dox has been reported to induce MMP-2 and MMP-9 in cardiac myocytes via MAP kinase pathway as well as through AP-1 pathway in oxidative stress [84, 85]. MMP-14 is a crucial player in active cellular invasion. Being a membrane-anchored protein, it localizes to the leading-edge plasma membrane where it catalytically activates all of the pro- (inactive) soluble MMP-2 and MMP-9 ref. [86]. Results from this study show sublethal Dox induces multiple soluble MMPs as well as the membrane-anchored MMP responsible for their activation and subsequent ECM remodeling; encompassing all of the major players in proteolytic-dependent metastatic phenomena. Taken together, these results further confirm the activation of an invasive program in MCF7 cells treated with sublethal dose of Dox. While it is important to verify the activation of known upstream signaling pathways in the induction of MMPs, the focus of the current study is understanding the deleterious effect of sublethal Dox on cancer. As such, MMP expression has served as additional marker for an invasive phenotype.

Dox is a DNA-damaging agent that sets in motion a signaling cascade known as the DNA Damage Response (DDR) [11, 12]. As many chemotherapies function by inducing DNA damage, it was important to determine if SFK induction is directly coupled to the DDR. Indeed, results showed that knockdown of p53 and ATR kinase—key effectors of the DDR response—were able to partly attenuate Dox induction of Fyn and Yes. It is not clear why the effect was specific to ATR and not ATM. It is reported that ATM is activated by double-strand DNA breaks while ATR is activated by broad spectrum DNA damages, including double-strand and single-strand breaks [87]. The downstream targets for ATM and ATR have broad overlap, yet these kinases also have distinct functions [12, 13]. These differences could be a factor in why SFK induction is affected by loss of ATR but not ATM. Notably, while there have been reports of ATR and p53 regulating Src phosphorylation [88], to our knowledge, the effect of ATR and p53 signaling on SFK at the message level has not been explored. Interestingly, Src has been shown to modulate both ATR and p53 activity [89, 90]. Despite these connections, several other agents that induce DNA damage and/or p53 expression had more modest effects on SFK expression, i.e., 2-3-fold for Fyn vs the 20-25-fold seen with Dox, and did not increase migration. Additionally, modest increases in Fyn expression by transient overexpression of p53 suggest that p53 augments but is not wholly sufficient to induce SFKs and likely requires other inputs. This was further supported by the blunted SFK induction that was observed in BC cell lines harboring p53 mutations. The involvement of p53-independent pathways likely specific to Dox may also offer an explanation for why other DDR-activating chemotherapeutic agents may not induce SFKs to the same extent. Our studies here suggest such pathways are independent of either Top2α or Top2β which are major mediators of the Dox response. Preliminary experiments with the antioxidant N-acetylcysteine also suggest that ROS generation does not contribute to SFK induction either (data not shown). These mechanisms and pathways are currently under further investigation.

## Conclusions

The results of this study demonstrate an undesirable pro-metastatic effect induced by sublethal Dox treatment of a non-invasive BC cell line. From a clinical perspective, while drug concentrations around the tumor maybe initially high, cancer cells in larger and/or poorly perfused tumors may be exposed to lower than effective dose of drug [91]. While such conditions can promote drug resistance [92], the results of our study suggest that in the context of Dox treatment, this could also increase the likelihood of local cancer cell invasion. Although this does not appear to be a general consequence of DDR activation by other chemotherapies, this emphasizes the importance of optimal dosing in Dox treatment. Furthermore, by identifying SFKs as mediators of the pro-migration/invasion effects of Dox, our results identify a therapeutic strategy to mitigate local invasion through co-treatment with Dasatinib.

## Supplementary Information

**Additional file 1: Supplemental Figure 1.** Cells resume growth after removal of Dox. MCF7 cells were seeded in 6-well plates and treated with vehicle (DMSO) or 0.4 μM Dox. After 24 h, cells were washed twice with 1XPBS and replaced with fresh media. Viable cell number was assessed by MTT at each time point shown (Mean ± SEM, *n* = 4).

**Additional file 2: Supplemental Figure 2.** Induction of Src Family Kinases by Dox treatment in MDA-MB-231 and SKBR3 cells. qRT-PCR analysis of Src, Fyn, and Yes was performed using actin as a reference gene in (A-C) MDA-MB-231; (D-F) SKBR3 cells. Data is presented as mean normalized expression (Mean ± SEM, * p < 0.05, ** p < 0.01 vs. vehicle, *n =* 3 for both cell lines).

**Additional file 3: Supplemental Figure 3.** Sublethal doses of Dox in ZR75-1, SKBR3 and U-2OS cells. Cells were treated with vehicle (DMSO) or Dox for the doses shown. Protein was extracted and immunoblotted for total PARP and Actin in (A) ZR75-1 (B) SKBR3 and (C) U-2OS cells.

**Additional file 4: Supplemental Figure 4.** Validation of Src Family Kinase induction by Dox treatment. Cells were treated with vehicle (DMSO) or Dox for the doses indicated; protein was extracted and immunoblotted for phospho-Src, Src, Yes and Actin in (A) MCF7; (B) ZR75-1; and (C) U-2OS cells.

**Additional file 5: Supplemental Figure 5.** Dox induction of Fyn is dependent on p53. ZR75-1 cells were treated with siRNA, vehicle (DMSO) and 0.4 μM Dox as shown; (A-C) Cells treated with AS negative control or p53 siRNA were analyzed for (A) protein by immunoblot for p53 and Actin; (B) Fyn; and (C) Yes expression by qRT-PCR analysis. Data is presented as mean normalized expression (Mean ± SEM, *p < 0.05 *n =* 3). MCF7 cells were transfected with empty vector (EV; pcDNA3) or wild-type p53 plasmid as shown; (D) protein was extracted and immunoblotted for p53, phospho-Src, Src, Yes and Actin; expression of (E) Src; (F) Fyn; and (G) Yes were analyzed by qRT-PCR. Data is presented as mean normalized expression (Mean ± SEM, *p < 0.05 *n =* 4).

**Additional file 6: Supplemental Figure 6.** Verification of knockdown of Fyn and Yes in transwell migration assay. MCF7 cells were treated with siRNA, vehicle (DMSO) and 0.4 μM Dox as shown. (A-C) Cells treated with AS negative control, Fyn, or Yes siRNA, were analyzed for (A) verification of knockdown of Fyn by qRT-PCR for Fyn; (B-C) verification of knockdown of Yes by (B) qRT-PCR and (C) immunoblot for Yes. Actin was used as a reference gene. Data presented as mean normalized expression (Mean ± SEM, *p < 0.05 vs. si-AS, *n =* 3).

## Data Availability

The data supporting the conclusions of this article is included within the article.

## Abbreviations

Veh: Vehicle
Dox: Doxorubicin
Das: Dasatinib
DMSO: Dimethyl sulfoxide
ROS: Reactive oxygen species
EMT: Epithelial to mesenchymal transition
CSC: Cancer stem cell
SFK: Src Family Kinase
DDR: DNA damage response
ECM: Extracellular matrix
LDH: Lactate dehydrogenase
MTT: (3-(4,5-Dimethylthiazol-2-yl)-2,5-diphenyltetrazolium bromide)
AS: Allstars negative control siRNA
ATM: Ataxia-telangiectasia-mutated
ATR: Ataxia-telangiectasia and Rad3-related
Top2α: Topoisomerase 2α
Top2β: Topoisomerase 2β
MMP: Matrix metalloproteinase
