# Supplementary figures and images for: Sublethal doxorubicin promotes migration and invasion of breast cancer cells: role of Src Family non-receptor tyrosine kinases
Source: Breast Cancer Res. 2021 Jul 27;23:76. doi: 10.1186/s13058-021-01452-5 (PMC8317414; doi:10.1186/s13058-021-01452-5)

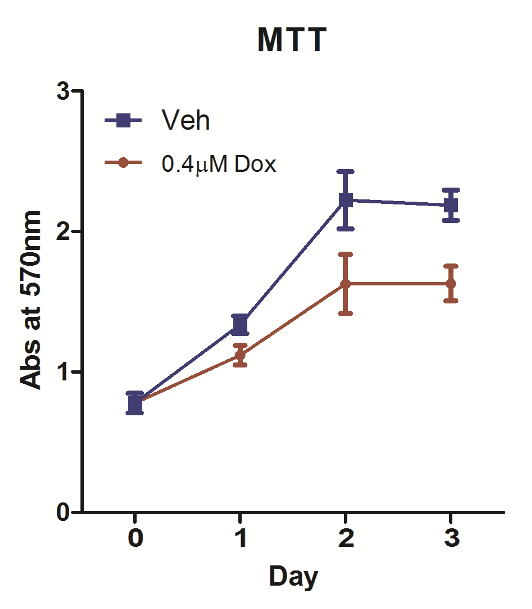

Supplement: Supplementary file 1 — Additional file 1: Supplemental Figure 1. Cells resume growth after removal of Dox. MCF7 cells were seeded in 6-well plates and treated with vehicle (DMSO) or 0.4 μM Dox. After 24 h, cells were washed twice with 1XPBS and replaced with fresh media. Viable cell number was assessed by MTT at each time point shown (Mean ± SEM, n = 4). [file 13058_2021_1452_MOESM1_ESM.tiff]

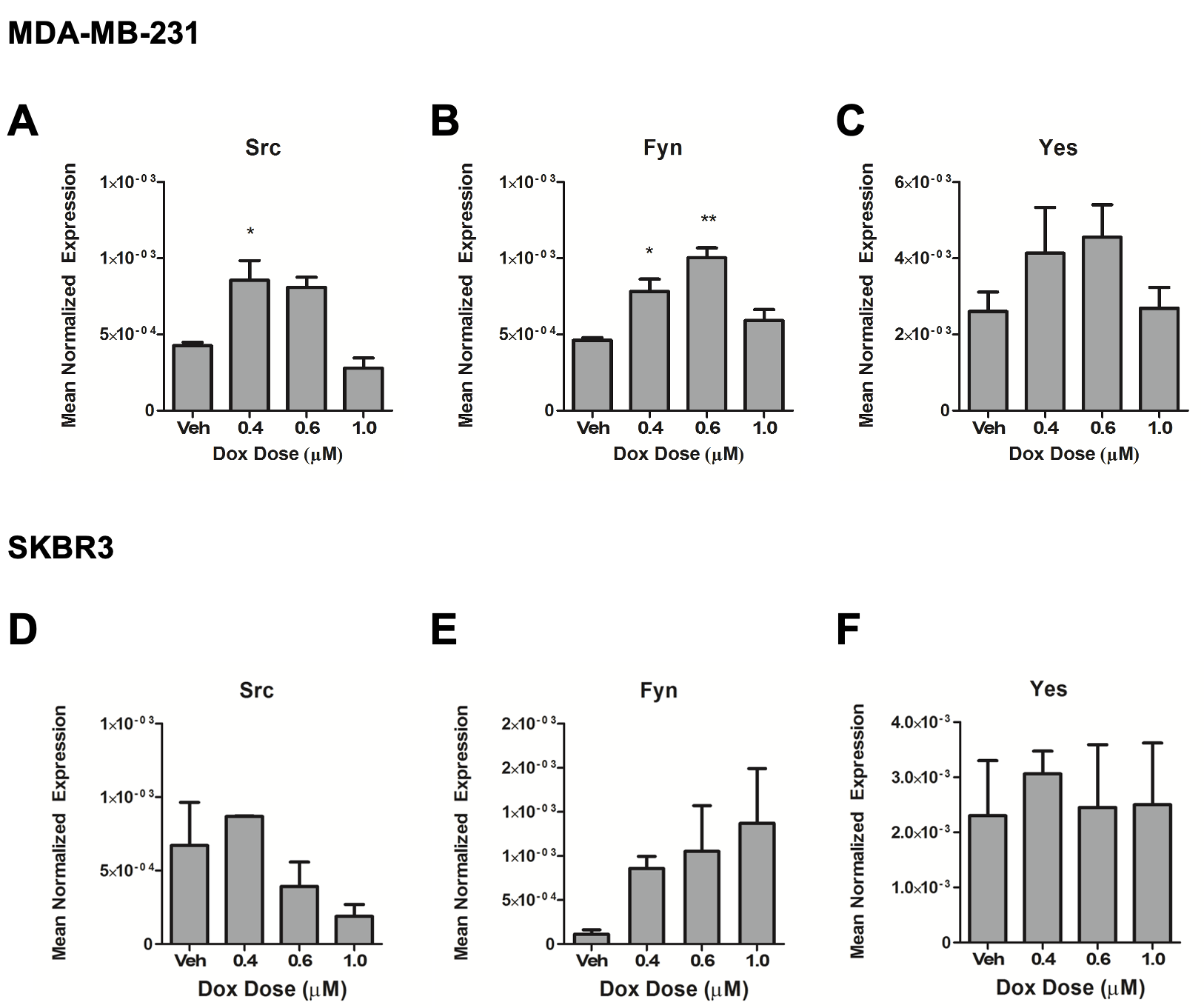

Supplement: Supplementary file 2 — Additional file 2: Supplemental Figure 2. Induction of Src Family Kinases by Dox treatment in MDA-MB-231 and SKBR3 cells. qRT-PCR analysis of Src, Fyn, and Yes was performed using actin as a reference gene in (A-C) MDA-MB-231; (D-F) SKBR3 cells. Data is presented as mean normalized expression (Mean ± SEM, * p < 0.05, ** p < 0.01 vs. vehicle, n = 3 for both cell lines). [file 13058_2021_1452_MOESM2_ESM.tiff]

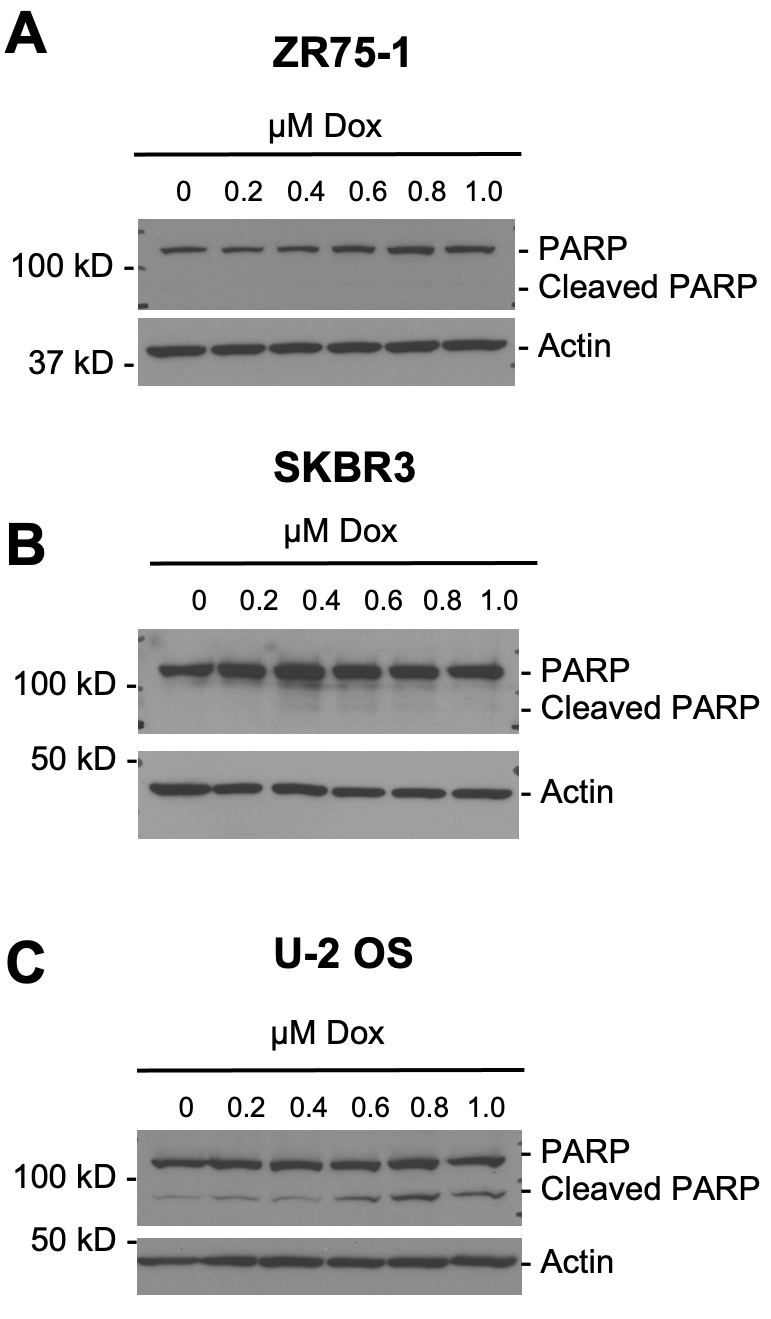

Supplement: Supplementary file 3 — Additional file 3: Supplemental Figure 3. Sublethal doses of Dox in ZR75-1, SKBR3 and U-2OS cells. Cells were treated with vehicle (DMSO) or Dox for the doses shown. Protein was extracted and immunoblotted for total PARP and Actin in (A) ZR75-1 (B) SKBR3 and (C) U-2OS cells. [file 13058_2021_1452_MOESM3_ESM.tiff]

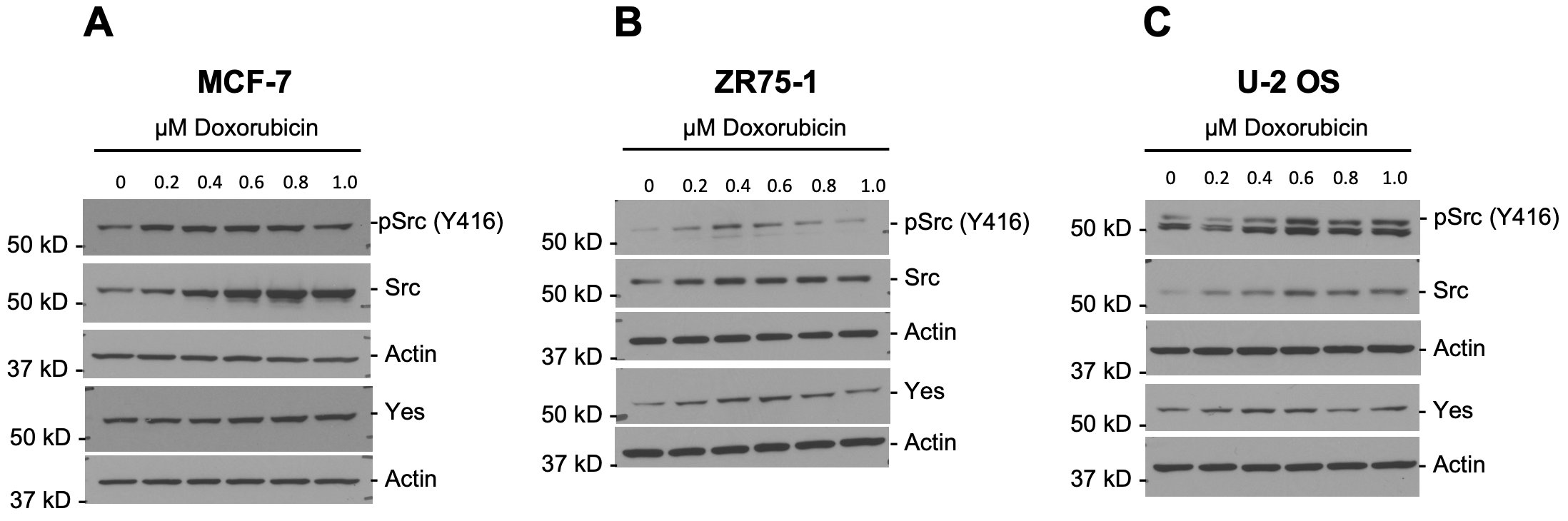

Supplement: Supplementary file 4 — Additional file 4: Supplemental Figure 4. Validation of Src Family Kinase induction by Dox treatment. Cells were treated with vehicle (DMSO) or Dox for the doses indicated; protein was extracted and immunoblotted for phospho-Src, Src, Yes and Actin in (A) MCF7; (B) ZR75-1; and (C) U-2OS cells. [file 13058_2021_1452_MOESM4_ESM.tiff]

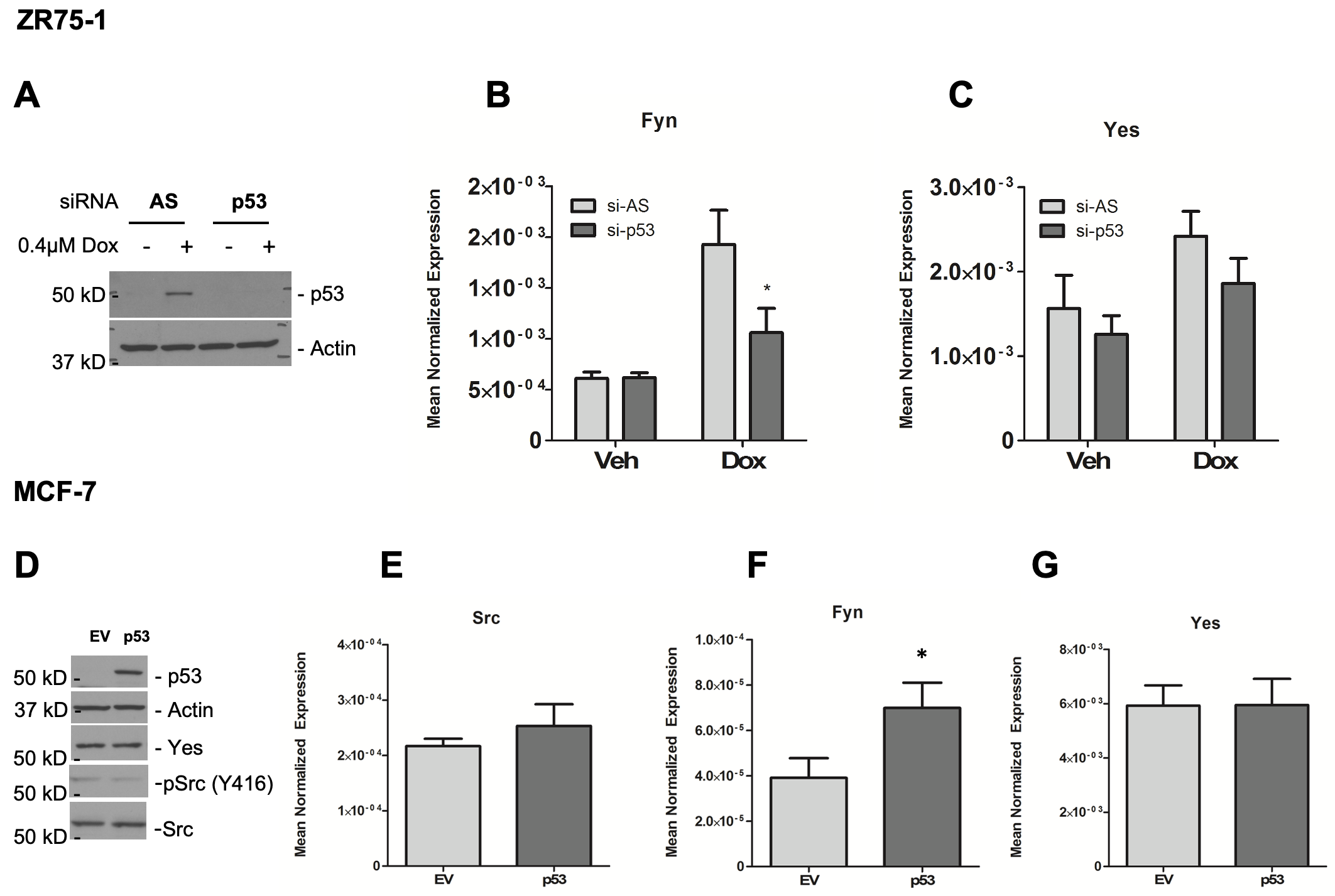

Supplement: Supplementary file 5 — Additional file 5: Supplemental Figure 5. Dox induction of Fyn is dependent on p53. ZR75-1 cells were treated with siRNA, vehicle (DMSO) and 0.4 μM Dox as shown; (A-C) Cells treated with AS negative control or p53 siRNA were analyzed for (A) protein by immunoblot for p53 and Actin; (B) Fyn; and (C) Yes expression by qRT-PCR analysis. Data is presented as mean normalized expression (Mean ± SEM, *p < 0.05 n = 3). MCF7 cells were transfected with empty vector (EV; pcDNA3) or wild-type p53 plasmid as shown; (D) protein was extracted and immunoblotted for p53, phospho-Src, Src, Yes and Actin; expression of (E) Src; (F) Fyn; and (G) Yes were analyzed by qRT-PCR. Data is presented as mean normalized expression (Mean ± SEM, *p < 0.05 n = 4). [file 13058_2021_1452_MOESM5_ESM.tiff]

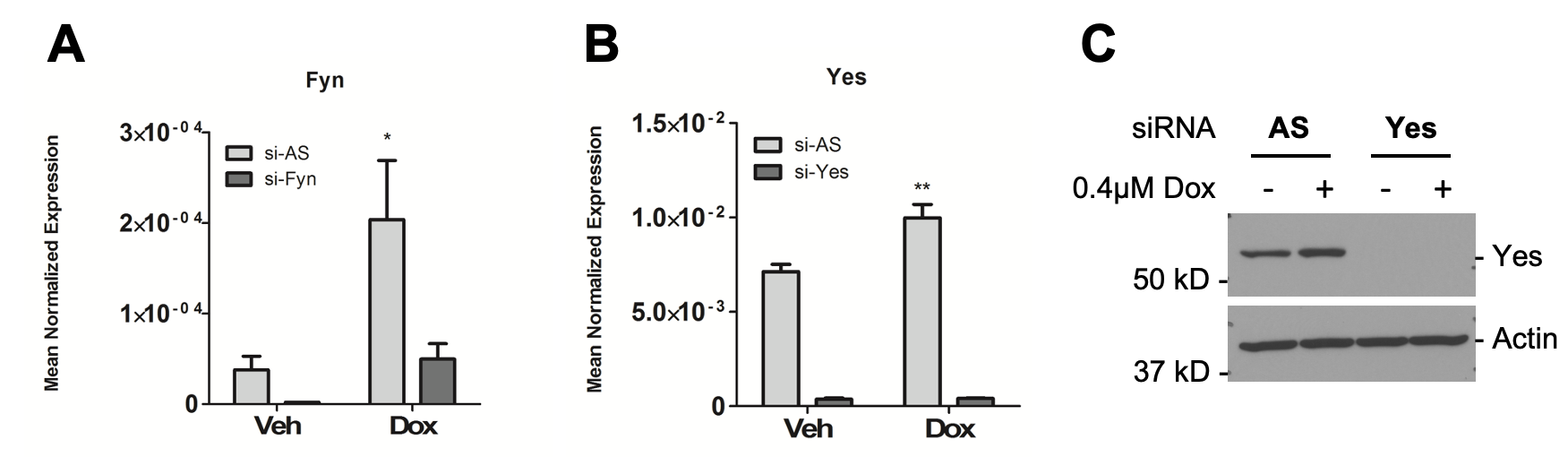

Supplement: Supplementary file 6 — Additional file 6: Supplemental Figure 6. Verification of knockdown of Fyn and Yes in transwell migration assay. MCF7 cells were treated with siRNA, vehicle (DMSO) and 0.4 μM Dox as shown. (A-C) Cells treated with AS negative control, Fyn, or Yes siRNA, were analyzed for (A) verification of knockdown of Fyn by qRT-PCR for Fyn; (B-C) verification of knockdown of Yes by (B) qRT-PCR and (C) immunoblot for Yes. Actin was used as a reference gene. Data presented as mean normalized expression (Mean ± SEM, *p < 0.05 vs. si-AS, n = 3). [file 13058_2021_1452_MOESM6_ESM.tiff]
